# Genomic comparison of *Planktothrix agardhii* isolates from a Lake Erie embayment

**DOI:** 10.1371/journal.pone.0273454

**Published:** 2022-08-23

**Authors:** Katelyn M. McKindles, R. Michael McKay, George S. Bullerjahn

**Affiliations:** 1 Great Lakes Institute for Environmental Research, University of Windsor, Windsor, ON, Canada; 2 Department of Ecology and Evolutionary Biology, University of Michigan, Ann Arbor, MI, United States of America; 3 Great Lakes Center for Fresh Waters and Human Health, Bowling Green State University, Bowling Green, OH, United States of America; 4 Department of Biological Sciences, Bowling Green State University, Bowling Green, OH, United States of America; University of Wisconsin Milwaukee, UNITED STATES

## Abstract

*Planktothrix agardhii* is a filamentous cyanobacterial species that dominates harmful algal blooms in Sandusky Bay, Lake Erie and other freshwater basins across the world. *P*. *agardhii* isolates were obtained from early (June) blooms via single filament isolation; eight have been characterized from 2016, and 12 additional isolates have been characterized from 2018 for a total of 20 new cultures. These novel isolates were processed for genomic sequencing, where reads were used to generate scaffolds and contigs which were annotated with DIAMOND BLAST hit, Pfam, and GO. Analyses include whole genome alignment to generate phylogenetic trees and comparison of genetic rearrangements between isolates. Nitrogen acquisition and metabolism was compared across isolates. Secondary metabolite production was genetically explored including microcystins, two types of aeruginosin clusters, anabaenopeptins, cyanopeptolins, microviridins, and prenylagaramides. Two common and 4 unique CRISPR-cas islands were analyzed for similar sequences across all isolates and against the known *Planktothri*x-specific cyanophage, PaV-LD. Overall, the uniqueness of each genome from *Planktothrix* blooms sampled from the same site and at similar times belies the unexplored diversity of this genus.

## Introduction

*Planktothrix agardhii* is a bloom-forming filamentous, non-diazotrophic cyanobacterium commonly inhabiting eutrophic freshwaters worldwide [1]. In North America, harmful algal blooms have been reported in temperate reservoirs and lakes [2–4]), and nearshore environments and estuaries in the Laurentian Great Lakes [5–7]. As an example, *P*. *agardhii* dominates the cyanobacterial community in Sandusky Bay, a drowned river mouth emptying into the open waters of Lake Erie [8]. Recent work has focused on the conditions favoring *P*. *agardhii* blooms over other bloom-forming taxa, such as *Microcystis* spp., that more commonly form HABs worldwide [9]. Prior work has shown that *P*. *agardhii* is well adapted to conditions of nitrogen deficiency that occur in the Bay as a consequence of denitrification [8, 10]. Combined with the observation that *P*. *agardhii* can scavenge regenerated ammonium more effectively than *Microcystis* spp. [11], and that this species can grow at a broad temperature range [12, 13], it has been proposed that *Planktothrix* blooms can form earlier in the spring than can *Microcystis* and then persist following the onset of summertime denitrification [8, 10, 11]. Despite this hypothesis, genetic analysis of local isolates had not yet been performed to test the diversity of nitrogen scavenging genes in *P*. *agardhii*.

Harmful algal blooms (HABs) typically produce a suite of secondary metabolites, also known as cyanotoxins, which have been linked to health risks in animals and humans [14, 15]. The most notable cyanotoxins produced by *P*. *agardhii* are the hepatotoxic microcystins (MCs). MCs are synthesized nonribosomally by an enzyme complex consisting of 9 or 10 genes, depending on the genus [16–18]. These complexes are responsible for the synthesis of the molecular core of all microcystin congeners that a species can produce [16], while the various domains within this complex determine the microcystin congeners being produced [19]. *P*. *agardhii* and *P*. *rubescens* harmful algal blooms tend to have more microcystin per unit of cyanobacterial biomass than blooms dominated by other microcystin producing species [20]. In addition to the production of microcystins, *Planktothrix* species can produce multiple other secondary metabolites, many of which are thought to be protease inhibitors [21]. Cyanopeptolins, also called oscillapeptins in *Planktothrix* species, are another class of nonribosomally-synthesized peptides which are found in several genera of cyanobacteria, all sharing the same basic domain structure while coding for unique tailoring genes [22]. Aeruginosins are another class of secondary metabolites that are produced using a nonribosomal peptide synthetase (NRPS) core. Further, Planktonic species of *Planktothrix* are also known to contain biosynthetic clusters of microviridin (*mdn*), prenylagaramide (*pag*), anabaenopeptin (*apn*), oscillatorin (*osc*), and microginin (*mic*) [23–26], producing anabaenopeptins B and E/F, microviridin I, prenylagarmide B, and variants of aeruginosins and cyanopeptolins [27]. Local isolates have been identified to produce demethylated MC-RR, demethylated MC-LR, and MC-YR [28], but have not been genetically characterized nor tested for the production of alternative secondary metabolites.

*Planktothrix agardhii* is also a host to a number of cyanophages, only one of which is readily characterized. PaV-LD is a podoviridae (a naked phage with no tail) isolated from Lake Donghu in China [29]. The phage does not cause complete lysis (rupture and death) of the host, indicating that the host may have some mechanism of phage resistance. One such mechanism is the presence of a CRISPR-cas system, common in cyanobacterial genera. The CRISPR-cas systems include the CRISPR (clustered regularly interspaced short palindromic repeats) array, a series of alternating direct repeat sequences and spacer sequences from bacteriophages and plasmids, and CRISPR associated genes (cas) [30–32]. The CRIPSR-cas system found within *Microcystis aeruginosa* has been used to describe host-parasite interactions as CRISPR loci are considered to provide records of past infections [33–35]. *Microcystis* encodes for a number of different CRISPR-cas subtypes, as determined by the sequence and classification of the cas genes, including subtypes I-A, I-D, III-A, and III-B [34, 36]. These subtypes contain identifiable spacer sequences matching the known *Microcystis*- specific cyanophage Ma-LMM01 in genomes from the Netherlands and Japan, indicating a wide dispersal of Ma-LMM01-like cyanophages [33]. Further, these spacer sequences have been used in conjunction with metagenome sequencing of local samples to identify cryptic novel cyanophages [35]. This type of analysis has yet to be done using other cyanobacteria species, including *P*. *agardhii*.

As a first step in understanding the physiological capabilities of *P*. *agardhii* with respect to nutrient acquisition (especially N assimilation) and secondary metabolite production (toxins, antifungals), we have sequenced all 20 *P*. *agardhii* genomes from Sandusky Bay described in our earlier reports [28]. All 20 strains are closely related, but distinct from one another due to high levels of genetic rearrangement. These differences are exemplified in the grouping of the sequences, and further supported through the varied presence of biosynthetic gene clusters for secondary metabolite production.

## Materials and methods

### Sandusky Bay isolate cultures

Sandusky Bay *Planktothrix agardhii* strains (Strain numbers 18XX) were isolated during the 2018 sampling season as previously described [28]. In brief, samples from each site were serially diluted until less than ten filaments remained in a well. Single filaments were pulled from the lowest dilution using a capillary tube and placed in a clean well containing Jaworski’s Medium (JM; ccap.ac.uk). Plates with single filaments were incubated for several weeks and were monitored by microscopy for growth and contamination from other phytoplankton. Successful isolates were scaled up and maintained in batch cultures. Isolates were confirmed to be *Planktothrix* sp. through morphological observation (no heterocysts nor akinetes, blue-green filaments without sheaths, long with no constrictions at cross-cell walls [37]) and PCR with *P*. *agardhii* specific PCR primers rpoC1_Plank_F271 (5′-TGTTAAATCCAGGTAACTATGACGGCCTA-3′) and rpoC1_P_agardhii_R472 (5′-GCGTTTTTGTCCCTTAGCAACGG-3′) [38].

*P*. *agardhii* 1024–1034 series were isolated from Sandusky Bay during summer 2016 by isolating individual filaments on agar as described previously [39]. Briefly 100 microliters of water sample were incubated in the middle of an agar plate (BG11 medium [40], 0.6% (w/v) Bacto Agar). Individual filaments tended to move out of the incubated sample by gliding resulting in self-purification from all other non-motile organisms. 10–20 individual filaments were cut out using a tiny micro spade under a dissecting microscope under sterile conditions and transferred to a new agar plate sealed with parafilm. After 1–2 months the clonal culture was transferred into fluid BG11 medium. Using established multi locus sequence analysis [1] all ten strains clustered in *P*. *agardhii* / *P*. *rubescens* phylogenetic lineage number 1 which is known from typically shallow lakes in the temperate zone of the Northern hemisphere [1].

Cyanobacterial strains were grown as unialgal, non-axenic batch cultures in JM. The cultures were maintained in 125 mL glass flasks at 22°C. Light was supplied by warm-white fluorescent tubes at a light-dark cycle of 12 h:12 h at a photosynthetic photon flux density (PPFD) of 10 μmol photons m^−2^ s^−1^.

### DNA preparation and extraction

DNA extractions were performed on late exponential growth cultures by filtering 10–15 mL culture onto 0.22 μm Sterivex cartridge filters (EMD Millipore, Billerica, MA). Sterivex filters were stored at -80°C until extraction with the DNeasy PowerWater Sterivex DNA Isolation Kit (Qiagen, Germantown, MD) following the manufacturer’s instructions. DNA quantity was checked using a Quantus Fluorometer (Promega, Madison, WI) and the associated QuantiFluor ONE dsDNA System kit (Promega, Madison, WI), per manufacturer’s instructions.

### Generating *Planktothrix* contig lists from metagenomes

DNA isolated from strains 1025, 1027, 1031, 1033, 1808–1810, and 1813 were sequenced at the University of Michigan Advanced Genomics Core (Ann Arbor, MI). DNA isolated from strains 1026, 1029, 1030, 1032, 1801, 1803–1807, 1811, and 1812 were sequenced at HudsonAlpha Institute for Biotechnology (Huntsville, AL). At both locations, staff performed sample QC, library generation, and ran samples on a NovaSeq 6000 Sequencing System (Illumina, San Diego, CA). The paired-end reads were 150 bp in length.

Metagenomics analysis was performed using the CLC Genomics Workbench v. 12.0.2 (Qiagen, Redwood City, CA). FASTA files were imported into CLC Genomics Workbench with the default quality settings following Steffen et al. [2]. Failed reads were discarded during import. Paired-end reads for both samples were trimmed for quality prior to being combined for assembly into contigs (Automatic word and bubble size were selected as well as a minimum length contig length of 2,000 bp) using CLC Genomics Workbench *de novo* assembly function that also mapped reads back to the generated contigs. Contigs were joined by mapping them to the reference genome *P*. *agardhii* NIVA-CYA 126/8 (NZ_CM002803) and its plasmids (NZ_CM002804 –NZ_CM002808). Joined and unjoined contigs were then analyzed via BLAST against *P*. *agardhii* NIVA-CYA 126/8 (NZ_CM002803) and its plasmids (NZ_CM002804 –NZ_CM002808), *P*. *agardhii* NIES-204 (AP017991) and its plasmids (AP017992 –AP017995), *P*. *agardhii* NIVA-CYA 15 scaffolds 1–3 (NZ_KE734694 –NZ_KE734696), and *P*. *agardhii* NIVA-CYA 56/3 scaffolds 1–16 and 20 (NZ_KE734722 –NZ_KE734737) including contigs 145 (NZ_AVFY01000117) and 158–160 (NZ_AVFY01000129—NZ_AVFY01000131). All positive contigs with a greatest bit score ≥ 1000 and a greatest identity % ≥ 90 were pulled to generate a contig list for each isolate. Contig hit outputs can be found in S1 Table.

### Annotation of *Planktothrix* genomes

The sequence list for each isolate was annotated using the Find Prokaryotic Genes 2.1 function within the Functional Analysis tool of the Microbial Genomics Module on the CLC Genomics Workbench. The model training was set to learn one gene model for each assembly, the minimum gene length was 100 bp, the maximum gene overlap was 50 bp, and the minimum score was 5.0. The genetic code was set to 11 Bacterial, Archaeal and Plant Plasmid. The output from this function was a sequence list with coding sequence (CDS) annotations.

The CDS annotated sequences were assigned functions based on Best DIAMOND Hit. To generate the DIAMOND protein reference database, UniProt Reference Clusters (UniRef50) version 2019_03 was downloaded to the CLC Genomics Workbench and indexed. UniRef50 is built by clustering UniRef90 seed sequences that have at least 50% sequence identity to, and 80% overlap with, the longest sequence in the cluster. The indexed database was then used to assign function to each CDS annotation using the Annotate CDS with Best DIAMOND Hit 0.4 function of the Functional Analysis tool of the Microbial Genomics Module, with an E-value limit of 0.001 and standard search sensitivity.

In addition to Best DIAMOND functional assignment, the sequence lists were separately assigned Protein Family domains (Pfam) and Gene Ontology (GO). Pfam-A v32 database was downloaded from EMBL-EBI through the Download Pfam Database 2.0 function. The GO database was downloaded through the Download GO Database 0.3 function, which generated the database from the 2019-07-01 GO release. The contigs were annotated with both the Pfam and GO functions using the Annotate CDS with Pfam Domains function, which used profiles gathering cutoffs and removed overlapping matches from the same clan Pfam parameters and the complete GO basic GO subset. Pfam hit outputs can be found in S2 Table.

To determine if there were potential contaminating genes present in each isolate genome, the CDS files were submitted to GhostKoala [41], a KEGG orthology and links annotation program. The database was selected for “genus_prokaryotes + family_eukaryotes.” Output included functional and taxonomic classification of recognized protein entries. Non-cyanobacterial gene classifications were added up and recorded in Table 1, while the taxonomic breakdown was listed as S3 Table.

**Table 1:** Genome characteristics for Sandusky Bay *Planktothrix agardhii* isolates and reference sequence *Planktothrix agardhii* NIVA_CYA 126/8.

*Planktothrix agardhii* designation	Total length (kbp)	No. contigs and scaffolds	G+C content (%)	N50 (kbp)	No. protein-coding sequences	No. of coding sequences attributed to non-cyanobacteria
NIVA_CYA 126/8	5045.9	6	39.6	4785.6	4532	23
Plk1025	4974.0	18	39.6	4291.3	4533	35
Plk1026	5422.1	74	39.5	4662.3	5387	47
Plk1027	5152.6	23	39.7	4046.3	5176	34
Plk1029	5147.2	8	39.6	4508.1	5133	41
Plk1030	5114.1	37	39.6	4710.1	5099	41
Plk1031	5046.1	31	39.6	4696.5	4571	32
Plk1032	4991.8	13	39.6	4684.6	4985	29
Plk1033	5349.1	191	39.4	4058.0	5537	66
Plk1801	4856.2	18	39.7	4235.1	4912	43
Plk1803	4991.9	22	39.7	3052.9	4981	33
Plk1804	4869.8	8	39.6	4539.4	4868	33
Plk1805	5039.6	12	39.6	4104.9	5055	36
Plk1806	4970.4	9	39.6	4590.0	4972	33
Plk1807	5429.1	72	39.5	4511.2	5360	42
Plk1808	4965.4	9	39.6	4701.5	4475	30
Plk1809	5656.3	20	39.6	4804.8	5114	28
Plk1810	4890.6	11	39.6	4267.2	4451	18
Plk1811	5092.8	16	39.9	3879.8	5347	105
Plk1812	5908.4	160	39.4	4397.2	5948	65
Plk1813	4957.6	15	39.6	4586.0	4502	34

### Whole genome analysis

Annotated *P*. *agardhii* scaffolds and contigs were then exported to Geneious Prime (Biomatters Ltd.) version 2020.2.3 as individual sample sequence lists. To reorder the contigs, each sequence list was whole genome aligned to the reference genome *P*. *agardhii* NIVA-CYA 126/8 and its plasmids. The alignment options used the MCM algorithm with automatically calculated seed weight and minimum Locally Collinear Blocks (LCBs) score and the gap alignment was performed using MUSCLE 3.6 [42]. Reordering of the sequences is required to prevent Mauve from assuming extra rearrangements are part of the sequence.

Once all sequences are sorted, they are whole genome aligned to each other using the progressive Mauve algorithm with automatically calculated seed weight and minimum Locally Collinear Blocks (LCBs) score and the gap alignment was preformed using MUSCLE 3.6 [42]. Each sequence list was treated as a single multiple-chromosome genome for comparison purposes which included plasmid sequences.

Whole genome alignments were exported from Geneious Prime to the CLC Genomics Workbench to generate images and comparison statistics. Average Nucleotide Identity Comparison (beta) 1.0 workflow was run with the minimum similarity fraction and the minimum length fraction set to 0.8. The output included a heatmap where the upper comparison was Average Nucleotide Identity (ANI) with a color concentration gradient set from 93–100% and the bottom comparison was Alignment Percentage (AP) with a color concentration gradient set from 20–100%. Additionally, the Average Nucleotide Identity Comparison was used to generate a set of whole genome phylogenetic trees from both AP and ANI calculations using Unweighted Pair Group Method with Arithmetic Mean (UPGMA) and Neighbor Joining (NJ).

These trees were used to organize the genomes into 4 groups, which were used in the CLC Genomics Workbench to identify unique genes in each grouping through the Differential Abundance Analysis function. Metadata was filled out for each functional abundance table associated with each of the individual genomes, including which phylogenetic branch they were in. This assignment allowed for comparison across groups (ANOVA-like) to identify specific genes functions that was dominant in each group. Output of the analysis included fold change, p-value, false discovery rate (FDR) p-value, and Bonferroni corrected p-value. Gene functions with undefined fold changes (not observed or underreported) were removed from the analysis.

The *P*. *agardhii* whole genome groupings were also used in Geneious Prime for re-alignment to generate genome rearrangement figures (S1 Fig). Sequences from each group were whole genome aligned as described above. Individual groupings allowed for a closer examination of genome block rearrangement between closely related isolates.

### Comparative alignment of housekeeping genes

To confirm relationships between *P*. *agardhii* isolates as described in the whole genome analysis, as well as to genetically confirm the relationship between these isolates and previously sequenced *Planktothrix* spp., a concatenated housekeeping gene phylogenetic tree was generated using *fts*Z, *gyrB*, *ntcA*, *rpoB*, and *rpoC1* [1, 8, 43, 44]. Individual gene alignments were performed on each housekeeping gene using Muscle 3.8.425 and included references from *P*. *agardhii* NIES-204, *Planktothrix rubescens* strain PCC 7821, *P*. *agardhii* NIVA-CYA 126/8, and *Planktothrix rubescens* NIVA-CYA 18 when available. The individual alignments were then combined using the Concatenate Sequences or Alignments tool in Geneious Prime. Finally, the phylogenetic tree was generated in Geneious Prime Tree Builder using the Jukes-Cantor genetic distance model and UPGMA Tree Build method.

### Identification and alignment of secondary metabolite biosynthetic clusters

The following secondary metabolite clusters were analyzed in Geneious Prime: aeruginosin, anabaenapeptin, cyanopeptolin, microcystin, microviridin, and prenylagaramide. Genes were queried using a BLAST search of previously published reference sequences using both full names and gene abbreviations, which were extracted as individual biosynthetic clusters. When available, these same genes were also extracted from reference sequences: *P*. *agardhii* NIES-204, *P*. *rubescens* strain PCC 7821, *P*. *agardhii* NIVA-CYA 126/8, and *P*. *rubescens* NIVA-CYA 18. Extracted sequences were aligned using Geneious Alignment, which automatically determined direction of sequences, preformed a global alignment with free end gaps and had a cost matrix of 70% similarity (IUB) (5.0/-4.5). Alignments were used to generate UPGMA trees using Jukes-Cantor genetic distance models. Branches were collapsed at a distance of 0.002 to denote similarity between isolate sequences. For which isolates were collapsed into each head sequence, see S4 Table.

To identify isolates that represent secondary metabolite production diversity, the secondary metabolite alignments were concatenated and used to generate a phylogenetic tree, again using the Geneious Prime UPGMA and Jukes-Cantor genetic distance models. To supplement the tree, a presence/absence table was also generated.

### CRSIPR-cas diversity and repeat sequences

CRISPR-Cas clusters were queried and extracted using both full names and gene abbreviations in Geneious Prime. Extracted regions were aligned to identify common and unique clusters across the isolates and reference sequences when available. Extracted regions were then grouped and analyzed using the web based CRISPRCasFinder [45] using preset parameters. File outputs included FASTA files of the CRISPR spacer sequences, FASTA files of the CRISPR direct repeats, and identification of Cas genes and Cas subtypes. The FASTA file of CRIPSR spacer sequences was then used in a BLASTn seach (NCBI) under preset parameters to identify sequence similarities to PaV-LD and other *Planktothrix* spp. reference sequences.

Cluster figures were generated by importing an example sequence of each group into SnapGene Viewer software (from Insightful Science; available at snapgene.com).

### Nutrient acquisition and metabolic pathways

Specific genes of interest were identified based on ongoing work in our lab examining carbon metabolism, nutrient acquisition, and stress responses, which included *nblA* (BBD52965.1, WP_042151427.1; [46, 47]), cyanophycinase *cphB* [48] and both cyanophycin synthetases *cphA1* (WP_042153347.1; [49]) and *cphA2* (WP_042156315.1; [11])), carbonic anhydrases (BBD56413.1, CAC5345616.1, WP_042155137.1) and bicarbonate transporters (WP_026796371.1, WP_026785781.1; [50]). These genes were aligned in Geneious Prime with related genes from reference genomes as described above in other sections.

## Results

### General genome characteristics of *Planktothrix agardhii* isolates

*P*. *agardhii* isolates taken from Sandusky Bay, Lake Erie in 2016 and 2018 were comparable to the reference sequence of *P*. *agardhii* NIVA_CYA 126/8 and its plasmids. Indeed, the average total length of the genomes and plasmids were only slightly higher than the reference sequence at 5,182.6 ± 325.7 kbp and contained slightly more protein-coding sequences at 4540.8 ± 207.2 cds (Table 1).

**Fig 1:**
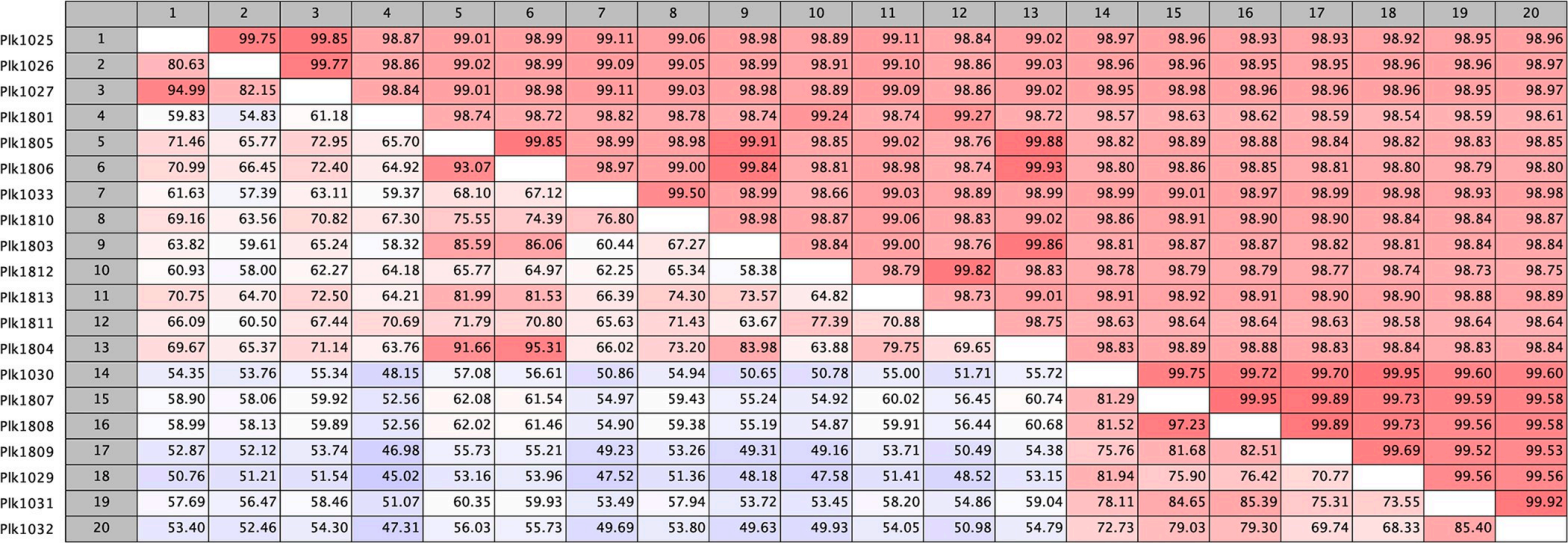
Relatedness of whole genome alignment of 20 *P*. *agardhii* isolates from Sandusky Bay, Lake Erie. The top of the matrix is the average nucleotide identity (ANI) common between two isolates. The bottom of the matrix is the alignment percentage (AP) common between two isolates. The lowest AP value suggests a common genome core of 45%.

**Fig 2:**
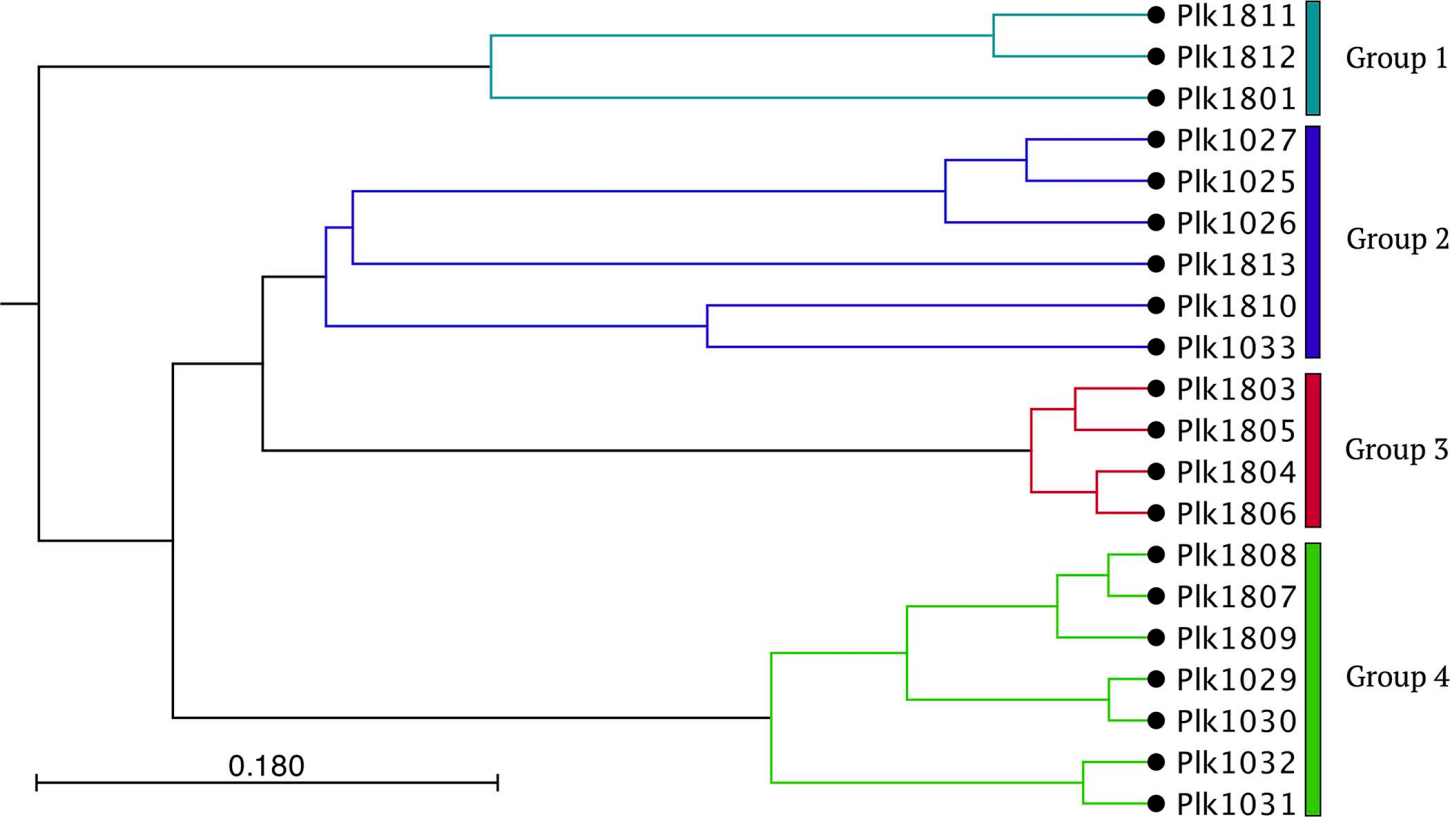
Whole genome phylogenetic tree based on (AP/ANI) reveals distinct grouping of *P*. *agardhii* isolates. Since the grouping is the same using either AP and ANI, only the tree generated using ANI and the UPGMA method is shown here. The bar represents the horizontal distance matrix used to scale the branch length as a function of substitutions per site.

When compared to each other, the Sandusky Bay isolate genomes have a high average nucleotide identity, which ranges from 98.54–99.95% (Fig 1). Alternatively, the genomes have a wide range of rearrangements, as determined by alignment percentages, which range from 45.02–97.23% (Fig 1). Since we were required to order the sequences according to a reference sequence during the generation of scaffolds and during the whole genome alignment process, the alignment percentage is a best approximation of genomic arrangement based on tools currently at our disposal. It is possible that through this manipulation, the Locally Collinear Blocks (LCBs) are spatially closer together, skewing the alignment percentage slightly higher. Note that the average nucleotide identity should not be significantly affected by this methodology. These measurements can be used to determine whole genome phylogenetic relationships to one another, clustering the isolates into 4 distinct groups (Fig 2).

These groups were then used to generate a Group Differential Gene Function table (Table 2) to determine if there were gene functional groups unique to specific linages of *P*. *agardhii*. These results are displayed as -fold changes compared to the other groupings combined and indicates an increased annotation of a specific gene functional group. Group 1 (denoted by the olive color in Fig 2) consists of *P*. *agardhii* 1811, 1812 and 1801. These isolates are characterized by increased glucose metabolism (GO:0005536 glucose binding at 3.08-fold more genes (p < 0.005) and GO:0051156 glucose 6-phosphate metabolism at 2.21-fold more genes associated with that group (p < 0.005)) and DNA maintenance (GO:0034061 DNA polymerase activity at 1.97-fold more genes (p < 0.001), GO:0004527 exonuclease activity at 1.56-fold more genes (p < 0.001), GO:0006260 DNA replication at 1.32-fold more genes (p < 0.001)) (Table 2). Group 2 (denoted by the orange color in Fig 2) consists of *P*. *agardhii* 1025, 1026, 1027, 1033, 1810 and 1813. These isolates are characterized by increased environmental response, including GO:0043571 maintenance of CRISPR repeat elements at 2.16-fold more genes associated with that group (p <0.001), GO:0009605 response to external stimulus at 1.88-fold more genes associated with that group (p < 0.005), and GO:0051704 multi-organism process at 1.65-fold more genes (p < 0.05). Group 3 (denoted by the green color in Fig 2) consists of *P*. *agardhii* 1803, 1804, 1805 and 1806. These isolates are characterized by increased metabolism, particularly GO:0016884 carbon-nitrogen ligase activity at 2.25-fold more genes (p < 0.05), GO:0016830 carbon-carbon lyase activity at 1.72-fold more genes (p < 0.01), GO:0009067 aspartate family amino acid biosynthetic process at 1.53-fold more genes (p < 0.005), and GO:1901361 organic cyclic compound catabolic process at 1.33-fold more genes associated with that functional group (p < 0.05). Group 4 (denoted by the blue color in Fig 2) consists of *P*. *agardhii* 1029, 1030, 1031, 1032, 1807, 1808 and 1809. These isolates are characterized by increased cellular respiration genes, most notable being GO:0070069 cytochrome complex at 4.51-fold more genes associated with that functional group (p < 0.001) and GO:004533 cellular respiration at 1.44-fold more genes associated with that functional group (p < 0.001).

**Table 2:** Group differential gene function table.

	GO function ID and Name	Log₂ fold change	Fold change	P-value	Bonferroni
Group 1: 1811 1812 1801	0005536 // glucose binding	1.63	3.08	1.9E-06	3.0E-03
	0051156 // glucose 6-phosphate metabolic process	1.15	2.21	2.3E-06	3.7E-03
	0034061 // DNA polymerase activity	0.98	1.97	0.0E+00	0.0E+00
	1990234 // transferase complex	0.82	1.77	0.0E+00	0.0E+00
	0016042 // lipid catabolic process	0.81	1.75	2.7E-05	4.0E-02
	0004527 // exonuclease activity	0.64	1.56	2.7E-11	4.2E-08
	0015666 // restriction endodeoxyribonuclease activity	0.56	1.47	1.9E-05	3.0E-02
	0006260 // DNA replication	0.4	1.32	2.2E-12	3.4E-09
	1902494 // catalytic complex	0.37	1.29	2.0E-10	3.2E-07
	0030234 // enzyme regulator activity	0.32	1.25	4.0E-08	6.3E-05
	0046983 // protein dimerization activity	0.29	1.23	2.8E-05	4.0E-02
	0016779 // nucleotidyltransferase activity	0.19	1.14	7.2E-07	1.1E-03
Group 2: 1025 1026 1027 1033 1810 1813	0016705 // oxidoreductase activity, acting on paired donors, with incorporation or reduction of molecular oxygen	1.66	3.16	0.0E+00	0.0E+00
	0008171 // O-methyltransferase activity	1.54	2.92	5.7E-10	9.0E-07
	0043571 // maintenance of CRISPR repeat elements	1.11	2.16	2.8E-10	4.5E-07
	0005506 // iron ion binding	1.03	2.04	0.0E+00	0.0E+00
	0009605 // response to external stimulus	0.91	1.88	7.4E-07	1.2E-03
	0051704 // multi-organism process	0.73	1.65	8.4E-06	1.0E-02
	0020037 // heme binding	0.66	1.58	1.7E-12	2.6E-09
	0046906 // tetrapyrrole binding	0.47	1.39	8.7E-12	1.4E-08
	0004519 // endonuclease activity	0.4	1.32	1.5E-06	2.4E-03
	0006304 // DNA modification	0.38	1.3	1.6E-10	2.6E-07
	0008170 // N-methyltransferase activity	0.34	1.27	3.0E-06	4.8E-03
	0046914 // transition metal ion binding	0.33	1.26	0.0E+00	0.0E+00
	0043414 // macromolecule methylation	0.33	1.25	6.3E-06	1.0E-02
	0006259 // DNA metabolic process	0.3	1.24	1.1E-14	1.8E-11
	0016758 // transferase activity, transferring hexosyl groups	0.28	1.22	2.3E-06	3.6E-03
	0016757 // transferase activity, transferring glycosyl groups	0.28	1.21	1.3E-12	2.0E-09
	0071840 // cellular component organization or biogenesis	0.19	1.14	2.7E-06	4.2E-03
	0008168 // methyltransferase activity	0.17	1.13	1.6E-05	3.0E-02
Group 3: 1803 1804 1805 1806	0016832 // aldehyde-lyase activity	1.93	3.8	2.0E-08	3.2E-05
	0016884 // carbon-nitrogen ligase activity, with glutamine as amido-N-donor	1.17	2.25	1.9E-05	3.0E-02
	0016830 // carbon-carbon lyase activity	0.78	1.72	4.8E-06	7.6E-03
	0009067 // aspartate family amino acid biosynthetic process	0.61	1.53	1.7E-06	2.6E-03
	0072330 // monocarboxylic acid biosynthetic process	0.51	1.42	2.7E-05	4.0E-02
	0030976 // thiamine pyrophosphate binding	0.49	1.4	1.5E-05	2.0E-02
	0034655 // nucleobase-containing compound catabolic process	0.48	1.39	4.2E-09	6.7E-06
	1901361 // organic cyclic compound catabolic process	0.41	1.33	1.4E-05	2.0E-02
	0046700 // heterocycle catabolic process	0.39	1.31	1.0E-05	2.0E-02
	0030259 // lipid glycosylation	0.38	1.3	7.3E-08	1.2E-04
	0016879 // ligase activity, forming carbon-nitrogen bonds	0.23	1.17	5.8E-07	9.2E-04
Group 4: 1029 1030 1031 1032 1807 1808 1809	0070069 // cytochrome complex	2.17	4.51	0.0E+00	0.0E+00
	0043565 // sequence-specific DNA binding	1.14	2.21	8.9E-16	1.4E-12
	0043531 // ADP binding	0.83	1.78	1.5E-08	2.4E-05
	0016763 // transferase activity, transferring pentosyl groups	0.59	1.5	9.0E-07	1.4E-03
	0045333 // cellular respiration	0.52	1.44	0.0E+00	0.0E+00
	0006400 // tRNA modification	0.51	1.42	2.3E-06	3.6E-03
	0004518 // nuclease activity	0.34	1.27	8.7E-06	1.0E-02
	0016788 // hydrolase activity, acting on ester bonds	0.32	1.25	3.6E-10	5.7E-07
	0006733 // oxidoreduction coenzyme metabolic process	0.3	1.23	6.4E-09	1.0E-05

**Fig 3:**
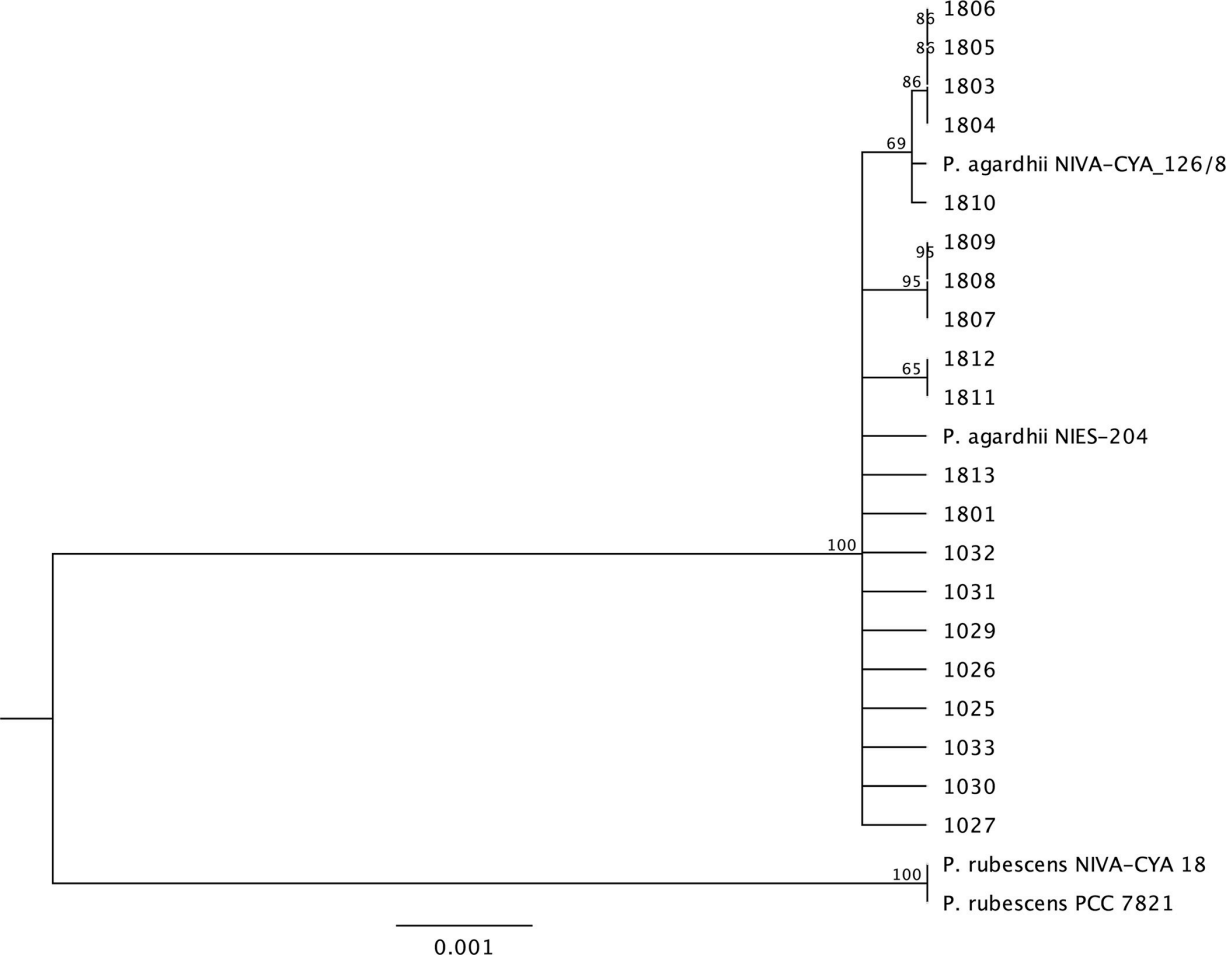
Concatenated conserved gene phylogenetic tree of *P*. *agardhii* isolates. Tree generated by concatenating the alignments of all Sandusky Bay isolates alongside two *P*. *agardhii* and two *P*. *rubescens* reference sequences. Genes included in concatenation include *ftsz*, *gyrB*, *ntcA*, *rpoB*, and *rpoC1*. The bar represents the horizontal distance matrix used to scale the branch length as a function of substitutions per site.

In addition to alignments, the genomes were analyzed based on a concatenation alignment of several housekeeping genes alongside reference sequences. All the Sandusky Bay isolates cluster together with *P*. *agardhii* NIVA-CYA 126/8 and *P*. *agardhii* NIES-204 and cluster separately from *Planktothrix rubescens* NIVA-CYA 18 and *Planktothrix rubescens* PCC7821 (Fig 3). Additionally, like the whole genome tree (Fig 2), Group 3 is still clustered together (1803, 1804, 1805, 1806) and Group 4 is clustered together (1029, 1030, 1031, 1032, 1807, 1808, 1809) (Fig 3). Groups 1 and 2 are not individually clustered in this initial analysis, likely representing relationships that can be described better using whole genome alignments as opposed to select housekeeping genes. In the same branch as Group 3, we have one reference sequence, *P*. *agardhii* NIVA-CYA 126/8, and the addition of *P*. *agardhii* 1810. As an outgroup for the *P*. *agardhii* isolates, we have *P*. *agardhii* 1033 and the second *P*. *agardhii* reference sequence, *P*. *agardhii* NEIS-204.

### Secondary metabolite biosynthetic clusters

**Fig 4:**
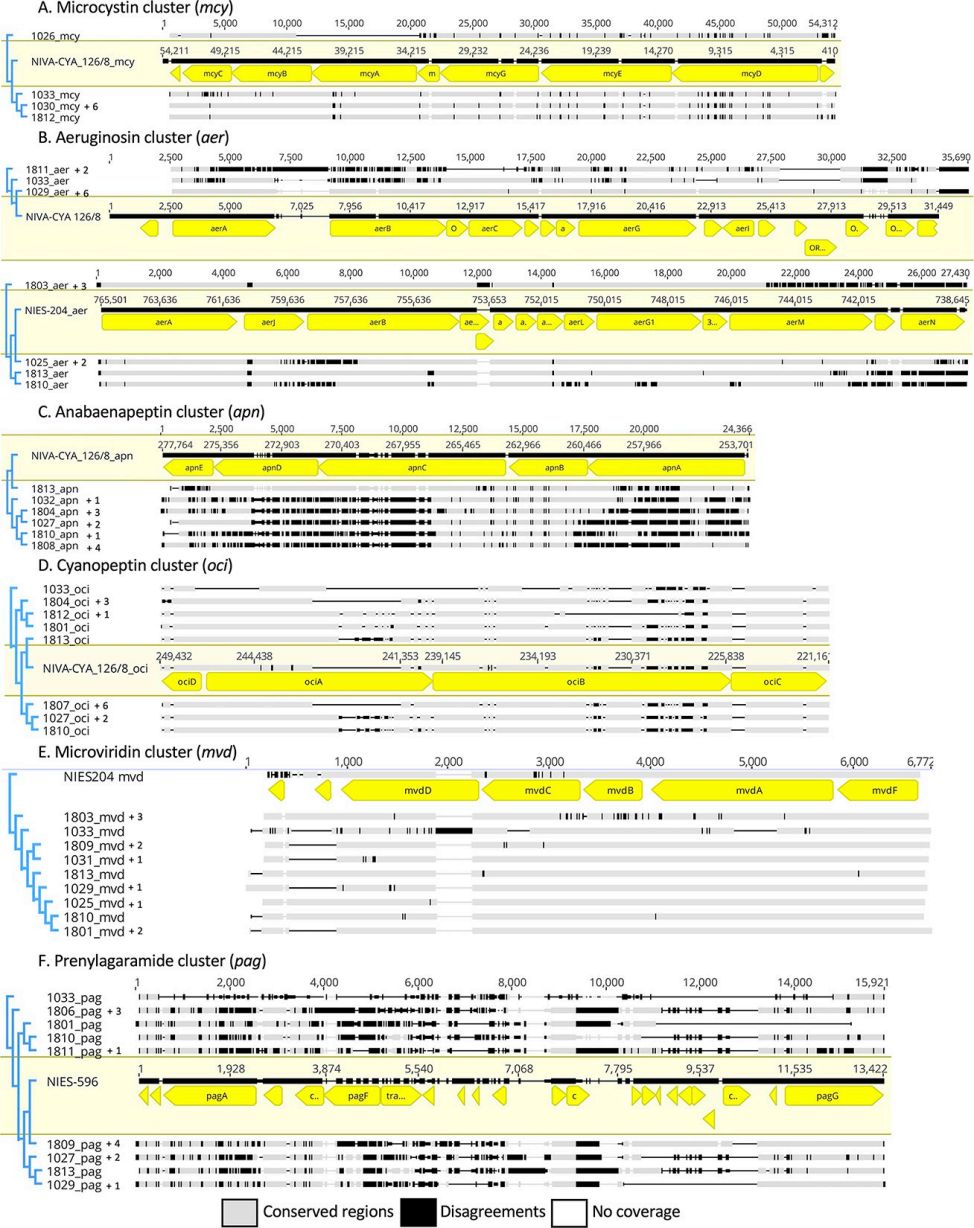
Alignments of unique secondary metabolite clusters as references for the relatedness of sequences between isolates. Reference sequence is highlighted in yellow and includes gene annotations for the clusters. Black segments in the non-highlighted sequences indicate points of difference, grey segments indicate similar regions, and the lines indicate regions of no coverage. A. Microcystin (*mcy*) cluster. B.Aeruginosin (*aer*) cluster. C. Anabaenapeptin (*apn*) cluster. D. Cyanopeptin (*oci*) cluster. E. Microviridin (*mvd*) cluster. F. Prenylagaramide cluster (*pag*). For which isolates were collapsed into each head sequence, see S4 Table.

Known secondary metabolite biosynthetic clusters which were found in the *P*. *agardhii* isolates include Microcystin (*mcy*), Aeruginosin (*aer*), Anabaenapeptin (*apn*), Cyanopeptolin (o*ci*), Microviridin (*mvd*), and Prenylagaramide (*pag*). At this time, no microginin gene cluster was identified. A full *mcy* cluster was found in isolates 1029, 1030, 1031, 1032, 1033, 1807, 1808, 1809, 1812, and a partial cluster was found in isolate 1026. The *mcy* clusters found in 1029, 1030, 1031, 1032, 1807, 1808, and 1809 were not genetically different, and were able to be collapsed into a single branch headed by 1030 (Fig 4A). Distinct from the rest of the full *mcy* cluster isolates is 1033, which contains mutations in *mcyC* and *mcyB* compared to the other isolates and the reference sequence (NIVA-CYA 126/8). Interestingly, isolate 1026 contains most of the genes of the *mcy* cluster, except for a deletion of *mcyA*. Two *aer* clusters were found in the different isolates, one set related to the biosynthetic cluster found in the reference NIVA-CYA 126/8 and one set related to the biosynthetic cluster found in the reference NIES-204 (Fig 4B). Eleven isolates contained the NIVA-CYA 126/8 biosynthetic cluster, including 1029, 1030, 1031, 1032, 1033, 1801, 1807, 1808, 1809, 1811, and 1812. Nine isolates contained the NIES-204 biosynthetic cluster, including 1025, 1026, 1027, 1803, 1804, 1805, 1806, 1810, and 1813. Seventeen isolates contained a heavily modified anabaenapeptin cluster, which collapsed into six distinct branches (Fig 4C). All 20 isolates contained a version of the cyanopeptin biosynthetic cluster (Fig 4D). Some clusters (branches headed by 1027, 1801, 1810, 1812, and 1813) were characterized by large insertion sequences in ociA, the nonribosomal peptide synthetase (NRPS) containing gene for this biosynthetic cluster. 19 of the isolates contained the microviridin biosynthetic cluster, which was relatively conserved across the sequences for genes *mvdA* and *mvdB*, and less so for *mvdC* and *mvdD* (Fig 4E). The least conserved biosynthetic cluster found in all 20 *P*. *agardhii* isolates was the biosynthetic cluster for Prenylagaramide (Fig 4F). This biosynthetic cluster is riddled internally with insertions and deletions, leaving the more conserved regions for the early and late portion of the cluster (*pagC*, *pagB*, *pagA*, and *pagG*).

**Fig 5:**
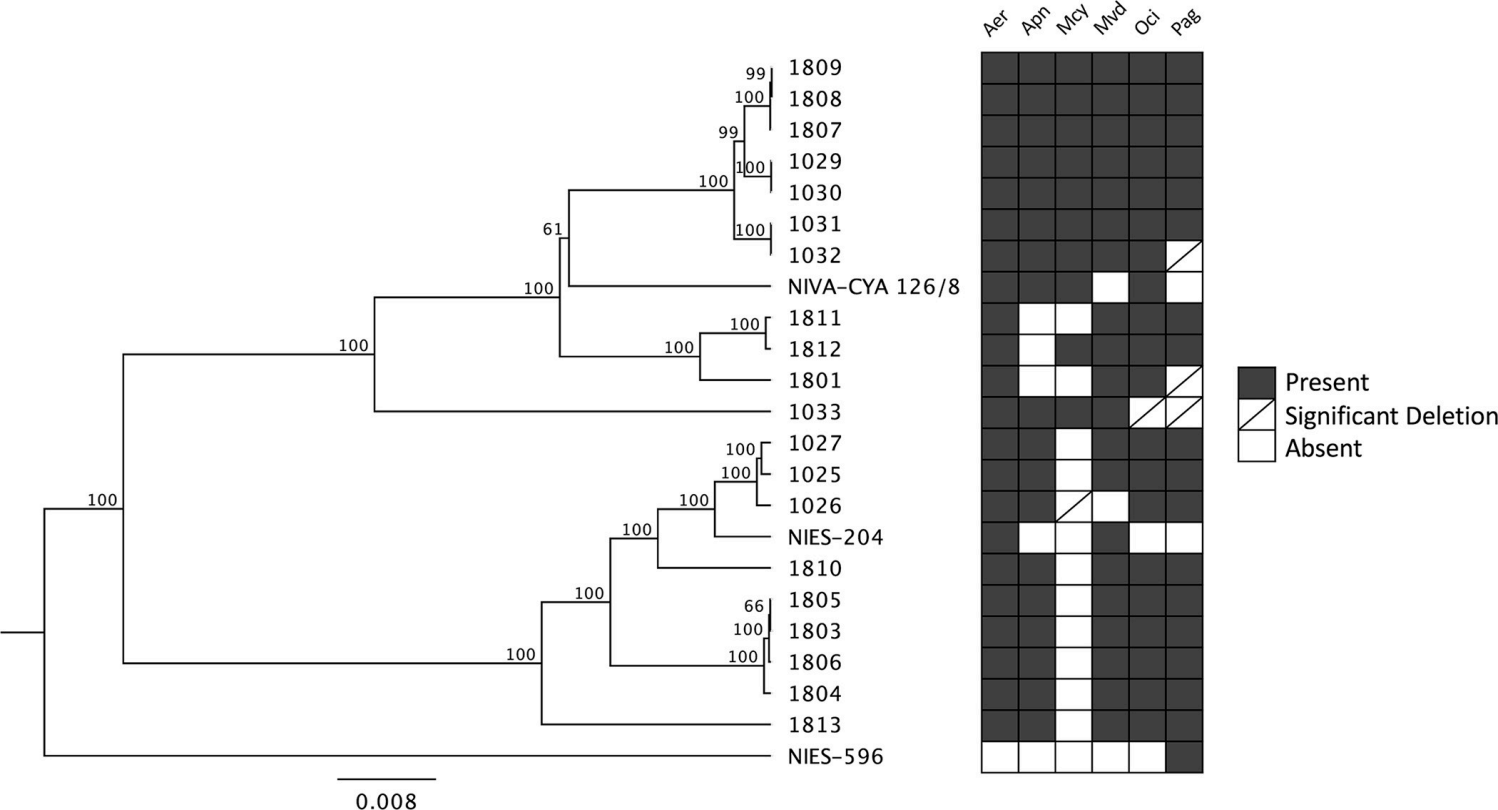
Oligotype phylogenetic tree, generated by the concatenation of the alignments for *mcy*, *oci*, *aer*, *apn*, *mvd*, and *pag*. The table relates presence and absence of specific secondary metabolite gene clusters to understand the relatedness of each isolate. The bar represents the horizontal distance matrix used to scale the branch length as a function of substitutions per site.

To identify particular isolates that represent secondary metabolite production diversity, the secondary metabolite alignments (Fig 4) were concatenated and used to generate a phylogenetic tree (Fig 5). Considerable similarity exists between some clusters, such as the non-mcy only cluster consisting of isolates 1803–1806, or the full suite cluster consisting of isolate 1029–1032 and 1807–1809. This analysis also identified several completely unique biosynthetic cluster sets in isolates 1033 and 1813, which were not driven by presence/absence alone.

### CRISPR-cas diversity and repeat sequences

**Fig 6:**
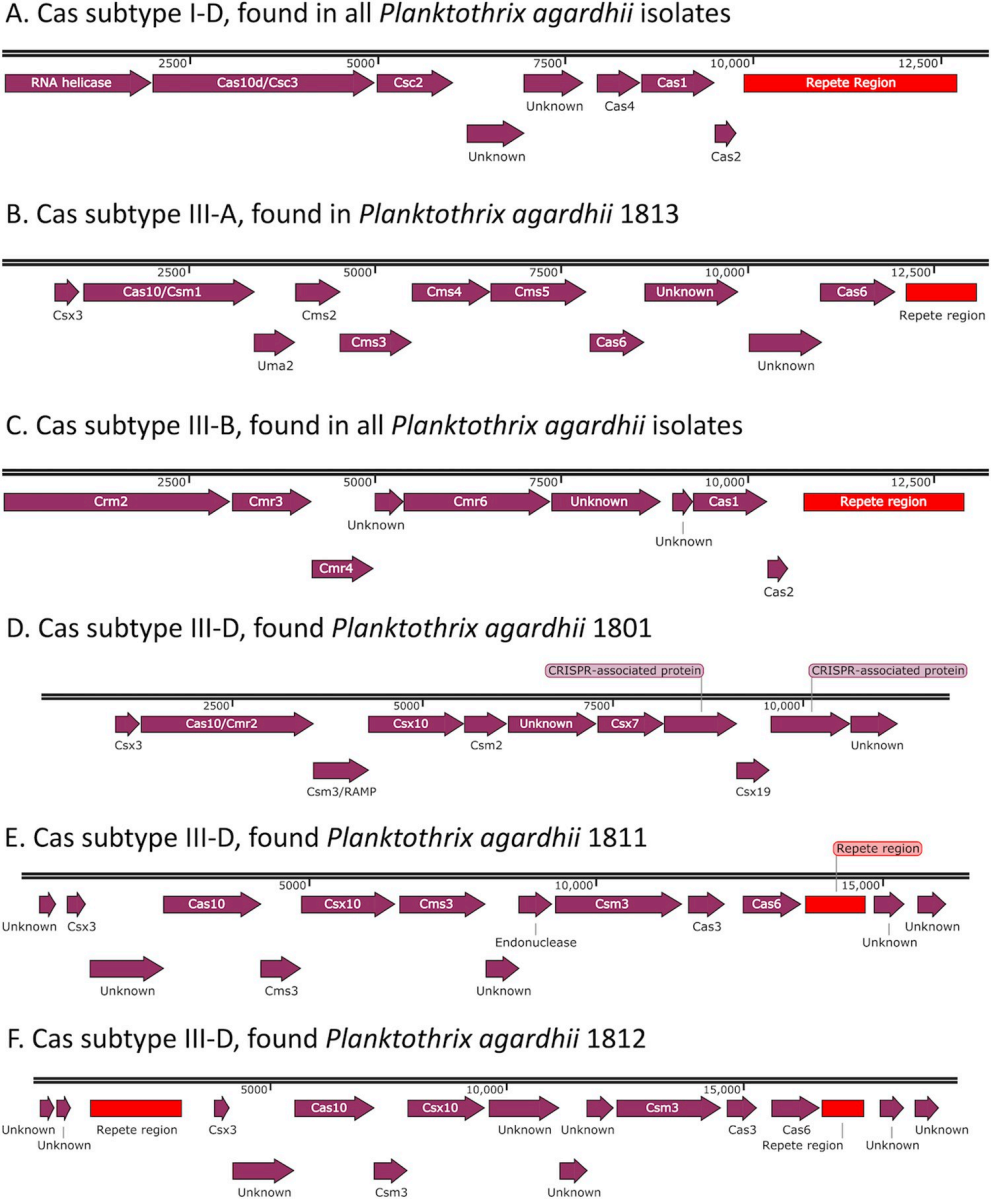
Common and unique CRISPR-Cas systems found in *P*. *agardhii* isolates of Sandusky Bay.

In an interest to identify pathogens that these isolates have encountered, the CRISPR-cas systems were analyzed, uncovering two common CRISPR-cas gene clusters across most isolate genomes, and four unique CRISPR-cas gene sets (Fig 6). The Cas subtype I-D (Fig 6A) is found in all the *P*. *agardhii* isolates, as well as in *P*. *agardhii* PCC 7805 and *P*. *agardhii* NIES-204. This cluster tended to be made up of 8 Cas genes and 18 spacer sequences with same direct repeat sequence (GTTTCAGTCCCGCAAGCAGGATTATTTTAATTGAAAG). The other common CRISPR-Cas system found in all the *P*. *agardhii* isolates was Cas subtype III-B (Fig 6C). This system was found in part within the reference sequences of *P*. *agardhii* PCC 7805 and *P*. *agardhii* NIES-204 but is missing the section from ~ 4000 to 9000 bp, including the genes Cmr4, Cmr6, and two genes of unknown function. The Cas subtype III-B cluster tended to be made up of 6–7 Cas genes and 23 spacer sequences with the same direct repeat sequence (GTTTCCAATCAATTAATTTCCCTAGCGAGTAGGGAG). Additionally, there were four Cas systems that were found only in a single *P*. *agardhii* isolate (Fig 6). In a BLAST search, none of these clusters showed greater than 35% similarity to any reference sequence. The first new CRISPR-Cas cluster, Cas subtype III-A (Fig 6B), was found in *P*. *agardhii* 1813. This cluster is made up of 7 Cas genes and 17 spacer sequences with the same direct repeat as the Cas subtype III-B cluster listed above. Next, we have three different Cas subtype III-D clusters, found in 1801 (Fig 6D), 1811 (Fig 6E), and 1812 (Fig 6F). The *P*. *agardhii* 1801 cluster is made up of 8 Cas genes, but contains no CRISPR arrays. The *P*. *agardhii* 1811 cluster consists of 6 Cas genes and a CRISPR array of 6 spacer sequences utilizing that same direct repeat (CTTTCAACTAATAGAATCCCGTTCGCGGGACTGAAAC). Finally, the *P*. *agardhii* 1812 CRISPR-Cas system is almost identical to the *P*. *agardhii* 1811 system, including the same number of Cas genes and same direct repeat sequence. The difference between the Cas subtype III-D in *P*. *agardhii* 1811 and 1812 is that there is a second CRISPR array in *P*. *agardhii* 1812 with a different repeat sequence (TGCAAAATGGGACACTTTGTAAA).

Given the general lack of cyanophage isolates and previous research stating that viral infections are common in cyanobacterial harmful algal blooms, the CRISPR arrays for each isolate was searched for viral sequences from the single *Planktothrix*-specific virus, PaV-LD (Table 3). Some open reading frames (ORFs) of PaV-LD appeared in several isolate CRISPR arrays, such as ORF007, which encodes a replicated DNA helicase, and ORF088, which encodes the tail tape measure protein. Of those that contained hits for ORF088, two sequences showed variability (1801_III-B_41 and 1811_I-D_27) which might suggest the presence of related, but not the same, *Siphoviridae*. Additionally, these viral sequences were found more frequently in *P*. *agardhii* isolate 1813 than in any other isolate (Table 3). FASTA sequences of each CRISPR array spacer can be found in the S5 Table.

**Table 3:** Table of CRISPR spacer sequences with matching PaV-LD ORF and function.

PaV-LD ORF	PaV-LD function	Lowest E-value	Greatest % Identity	Greatest Bit Score	CRISPR spacer
PaVLD_ ORF007R	replicative DNA helicase	5.95E-08	93.182	67.1	1025_III-B_24, 1026_III-B_17, 1027_III-B_24, 1029_III-B_32, 1031_III-B_30, 1032_III-B_32, 1807_III-B_32, 1808_III-B_30, 1809_III-B_30, 1813_III-A_48
PaVLD_ ORF088R	tail tape measure protein	8.26E-14	100	86	1029_III-B_31,1030_III-B_26, 1031_III-B_29, 1032_III-B_31, 1801_III-B_41*, 1807_III-B_31, 1808_III-B_29, 1809_III-B_29, 1811_I-D_27*, 1813_III-A_47
PaVLD_ ORF114L	hypothetical protein	2.81E-10	100	73.4	1029_III-B_22, 1030_III-B_19, 1031_III-B_20, 1032_III-B_22, 1807_III-B_22, 1808_III-B_20, 1809_III-B_20, 1811_III-B_41*, 1812_III-B_14*, 1813_III-A_39
PaVLD_ ORF027L	hypothetical protein	9.19E-11	100	75.2	1029_III-B_26, 1030_III-B_23, 1031_III-B_25, 1032_III-B_26, 1807_III-B_26, 1808_III-B_24, 1809_III-B_25, 1813_I-D_6*, 1813_III-A_43
PaVLD_ ORF119L	crossover junction endo-deoxyribonuclease	8.31E-08	100	64.4	1029_III-B_24, 1030_III-B_21, 1031_III-B_23, 1032_III-B_24, 1807_III-B_24, 1808_III-B_22, 1809_III-B_23, 1813_III-A_41
PaVLD_ ORF018R	integrase	2.38E-08	100	66.2	1029_I-D_9, 1030_I-D_9, 1031_I-D_9,1032_I-D_9, 1807_I-D_9, 1808_I-D_9, 1809_I-D_11
PaVLD_ ORF071R	capsid protein	0.002	93.75	50	1801_III-B_37*, 1803_I-D_4 1804_I-D_4, 1805_I-D_4, 1806_I-D_4
PaVLD_ ORF005R	replication-related protein	4.35E-07	94.872	62.6	1813_I-D_16
PaVLD_ ORF006R	hypothetical protein	2.98E-08	100	66.2	1813_I-D_1
PaVLD_ ORF010R	site-specific DNA methylase	2.38E-08	100	66.2	1813_I-D_22
PaVLD_ ORF056L	hypothetical protein	6.23E-08	100	64.4	1813_I-D_17
PaVLD_ ORF100R	anti-repressor protein	5.25E-04	91.667	52.7	1813_I-D_15
PaVLD_ ORF109R	hypothetical protein	2.90E-07	97.297	63.5	1813_I-D_13

*Denotes sequences with minor deviations from the other sequences for that PaV-LD ORF.

While some of the CRISPR array spacer sequences can be linked to PaV-LD, most of the sequences code for unknown organisms. Indeed, only 28.4% of the CRISPR array spacer sequences can be aligned with reference sequences; 13.4% can be found in *P*. *agardhii* NIES-204, *P*. *agardhii* PCC 7805, or *P*. *rubescens* PCC 7821, and 14.9% can be found in PaV-LD. There were four CRIPSR array spacer sequences which were found in half or more of the *P*. *agardhii* Sandusky Bay isolates (Table 4). The first spacer sequence can be found in 16 isolates, as well as *P*. *agardhii* NIES-204 and *P*. *agardhii* PCC 7805, suggesting common infectious agent across geographical distances (Table 4). The last two spacer sequences can be found in 10 and 9 isolates, respectively, and do not have any known reference sequence, likely denoting local infectious agents.

**Table 4:** Table of common CRISPR spacer elements across a majority of isolates (≥ 10).

CRISPR Spacer sequence:	Found in isolates:	Reference sequences (E-value)
TATTGCAAAACATTTACGATAGATAAAAAAACATTTTCT	1025, 1026, 1027, 1029, 1031, 1032, 1033, 1803, 1804, 1805, 1806, 1807, 1808, 1809, 1810, 1813	P. agardhii NIES-204 (8E-10) P. agardhii str. 7805 (8E-10)
AGGGAACTGCTATGTTTTTACCTCCTATGCGGTCATTACTTTTAA	1025, 1026, 1027, 1029, 1031, 1032, 1807, 1808, 1809, 1813	P. agardhii str. 7805 (9E-13)
TCGTTTTCAGCTTTTAATTTTTGGGCTTTTTTCTTGATTTCGTT	1025, 1026, 1027, 1029, 1031, 1032, 1807, 1808, 1809, 1813	None
CATAACTATTAACTATAGCAGTTTTTTCCTGTTCTT	1025, 1027, 1029, 1030, 1031*, 1032, 1807, 1808, 1810	None

*Denotes the presence of more than one copy of this spacer in different CRISPR segments.

### Nutrient acquisition and metabolic pathways

**Fig 7:**
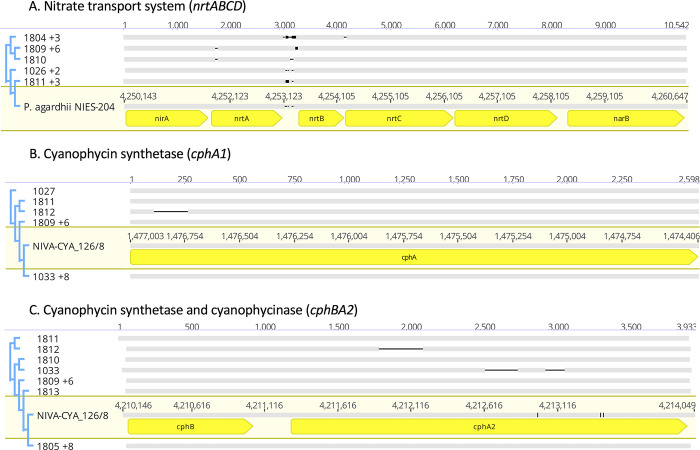
Nitrogen acquisition and storage genes found in *P*. *agardhii*. A. Sequence alignment of the *nrtABCD* cluster in reference NIES-204 and the *P*. *agardhii* isolates from Sandusky Bay. B. Sequence alignment of cyanophycin synthetase *cphA1*. C. Partial sequence alignment of cyanophycinase (*cphB*) and cyanophycin synthetase *chpA2* operon.

Given the hypothesis that *Planktothrix agardhii* dominates in some regions because it is a better scavenger for nitrogen, we analyzed the isolate genomes for several nitrogen metabolism genes and related them to reference sequences containing the same genes. First, we looked at the *nrtABCD* cluster, which encodes for a nitrate transport system, and its flanking genes, *narB*, which converts nitrate to nitrite, and *nirA*, which converts nitrite to ammonia (Fig 7A). This cluster was found in reference *P*. *agardhii* NIES-204, which showed sequence similarity to the cluster found in the isolates ranging from 97.2–99.9% identical. The most conserved genes compared to the reference were *nirA* and *nrtD*, while the least conserved genes were *nrtA*, *nrtB*, and *narB*. Indeed, the most common cluster among the isolates was the sequence found in 1809 (Fig 7), which was highly divergent in *nrtAB* and to a lesser degree in *nrtC*.

Several other genes included in the KEGG pathway for nitrogen metabolism were analyzed. In addition to the *nrtABCD* cluster as described above, there was the presence of an ABC-type nitrate/sulfonate/bicarbonate transporter (a *NitT/TauT* family) that was unique to three isolates and one reference sequence: NIES-204, 1025, 1026, 1027 (Table 5). Further, there are two ammonium transporters, *amt1* and *amt3*, which can be found in all isolates and both NIES-204 and NIVA-CYA 126/8 (Table 5). Sequence similarity was generally > 99% compared to reference sequences, apart from 1033 (96.1%) and 1813 (93.6%). Finally, there were several distinct beta carbonic anhydrases (CA) / carbonate dehydratase, which are involved in the conversion of HCO^3-^ to CO_2_. CA1 showed high conservation across the isolates and > 99.5% sequence similarity to the reference sequence. CA2 was also highly conserved, showing slightly lower sequence similarity to the reference at > 98.3%, but was missing from isolate 1812. CA3 was missing from three isolates: 1025, 1026, and 1027. These three isolates instead contained the carbonate dehydratase found in reference NIES-204 (Table 5).

**Table 5:** Sequence similarity of important nutrient acquisition genes for *Planktothrix agardhii*. Ammonium transporter genes are linked in the genome and were analyzed as a gene set.

	ABC-type nitrate/sulfonate/bicarbonate transporter	Ammonium transporters (amt1, amt3)	Carbonic anhydrase 1 (beta)	Carbonic anhydrase 2 (beta)	Carbonic anhydrase 3 (beta)	Carbonate dehydratase (beta)
NIES-204	Ref. (BBD53028.1)	-	Ref. (BBD56413.1)	Ref. (BBD55070.1)	-	Ref. (BBD56294.1)
NIVA-CYA 126/8	-	Ref. (WP_042151837.1, WP_072005174.1)	-	-	Ref. (WP_042155137.1)	-
1025	100	99.44	100	99.72	N/A	100
1026	100	99.42	100	99.72	N/A	100
1027	100	99.44	100	99.72	N/A	100
1029	N/A	99.93	99.66	99.86	100	N/A
1030	N/A	99.93	99.66	99.86	100	N/A
1031	N/A	99.93	99.83	99.72	100	N/A
1032	N/A	99.93	99.83	99.72	100	N/A
1033	N/A	96.11	99.83	99.72	99.85	N/A
1801	N/A	99.46	99.49	98.44	99.56	N/A
1803	N/A	99.46	99.83	98.3	100	N/A
1804	N/A	99.44	99.83	98.3	100	N/A
1805	N/A	99.46	99.83	98.3	100	N/A
1806	N/A	99.44	99.83	98.3	100	N/A
1807	N/A	99.1	99.83	99.86	100	N/A
1808	N/A	99.11	99.83	99.86	100	N/A
1809	N/A	99.11	99.83	99.86	100	N/A
1810	N/A	99.51	99.83	98.44	99.41	N/A
1811	N/A	99.13	99.49	98.3	99.56	N/A
1812	N/A	93.58	99.49	N/A	99.56	N/A
1813	N/A	99.42	99.83	99.57	100	N/A

Nitrogen storage and usage within the cell was examined by looking at the cyanophycin storage genes (*cphB*, *cphA1* and *cphA2*) and the phycobilisome degradation gene (*nblA*). *NblA* was 100% identical to the long *nblA* gene found within reference NIES-204 (protein ID: BBD52965.1) and NIVA-CYA 126/8 (protein ID: WP_027255584.1). Alternatively, there were differences in the *cphBA2* and *cphA1* genes between the Sandusky Bay isolates and the references (Fig 7B, 7C).

## Discussion

Here we present 20 isolates of *Planktothrix agardhii* isolated from the same geographical region (Sandusky Bay, Lake Erie) in two different bloom seasons: 2016 and 2018. These isolates have been sequenced and characterized in terms of relatedness to each other, production of secondary metabolites, CRISPR-cas defense system, and nutrient acquisition. These isolates are related but unique and aligned with the two reference sequences previously published. All the isolates from Sandusky Bay clustered with *P*. *agardhii* NIES-204, a strain from Lake Kasumigaura, Japan [51], and *P*. *agardhii* NIVA 126/8, a strain from Lake Langsjön, Sweden [39], separated from two *P*. *rubescens* strains (Fig 3), similar to the relationship seen in other studies [52]. Despite the difference in temporal isolation, these isolates share a minimum genomic core of 45% (Fig 1), and clustered in groups independent of year of isolation (Fig 2). This seems to reflect what is found in other cyanobacteria species in the Laurentian Great Lakes region, as work on Lake Erie *Microcystis* spp. identified a core genome of similar size at 45% [36].

The clusters reflect minor differences in metabolic processes (Table 2), suggesting that within the same population, these minor differences could be utilized for ecophyisological adaptations. Group 1 was characterized by increased gene presence related to glucose binding, which may allow for increase rates of uptake of organic carbon, which was shown to be low in *Planktothrix* under normal conditions [53]. Group 2 was characterized by an increased gene presence related to oxidoreductase activity, possibly indicating strains that are more efficient at cellular respiration, or better under stressful environments, as seen in *Microcystis* [54]. Group 3 was characterized by containing more genes associated with aldehyde-lyase activity, which may indicate elevated levels of amino acid biosynthesis and nutrient metabolism, particularly under self-shading or darker water conditions [53]. The last group was characterized by more cytochrome complex genes, possibly indicating isolates with increased photosynthetic capabilities [55].

Our *P*. *agardhii* isolate genomes contain multiple secondary metabolite biosynthetic clusters which are found in other isolates of the same species, including microcystins, two types of aeruginosin clusters, anabaenopeptins, cyanopeptolins, microviridins, and prenylagaramides. Previous characterization of some of these isolates have identified three microcystin congeners that are produced by them; demethylated MC-RR, demethylated MC-LR, and MC-YR [28]. Our genetic analysis of the MC biosynthetic cluster revealed the presence of a common cluster across 7 of the 10 MC-producing isolates (Fig 4A), which consisted of several dissimilar regions compared to the MC cluster found in reference *P*. *agardhii* NIVA-CYA 126/8, a strain capable of producing MC-RR and MC-LR [17]. This reference strain is also known to produce aeruginosins, anabaenopeptins and microviridins, all biosynthetic clusters that can be identified in the Sandusky Bay isolates (Fig 4B, 4C, 4E). Indeed, we required two reference sequences for the aeruginosin biosynthetic cluster (Fig 4B), as there are two distinct clusters which have been identified [56]. One or the other of these different but related clusters can be found in all the Sandusky Bay isolates. The cluster found in *P*. *agardhii* NIVA-CYA 126/8 is known to produce aeruginoside 126A and aeruginoside 126B (Ishida et al. 2007), while the cluster found in *P*. *agardhii* NIES-204 was thought to produce aeruginoside 102 based on its similarity to the clusters found in *Microcystis* NIES-843 [57] but may not produce aeruginosins at all due to the divided structure of aerK [56]. Unfortunately, full secondary metabolite screening has not yet been performed on these isolates, therefore we can only describe the genetic potential and not the actual production of any one secondary metabolite and its benefit to the producer.

This work presents the first analysis of the types of CRISPR-cas subtypes found in *P*. *agardhii* (Fig 6). The subtypes described here are not unique to *P*. *agardhii* as a majority of studied cyanobacterial genomes contain a subtype I-D system, which seems to be unique to the phylum *Cyanobacteria*, and subtypes III-A and III-B are rarer [58]. Indeed, much work has been done on the diversity of CRISPR-cas systems found in *Microcystis aeruginosa*, both locally [36] and abroad [34, 59]. These studies focus on the diversity of CRISPR spacer sequences, suggesting that these organisms are challenged by a diverse group of cyanophages and foreign DNA that are largely uncharacterized [33, 35]. The CRISPR spacer sequences described here (Table 3) for *P*. *agardhii* can be attributed to the single sequenced *Planktothrix*-specific cyanophage PaV-LD [29]. Nonetheless, these viral spacer sequences are only 14.9% of the CRISPR-cas system, meaning most of these sequences encode for unknown cyanophages and foreign plasmids. Interestingly, some CRISPR spacer sequences can be found in reference sequences of *P*. *agardhii* (Table 4), further suggesting that some foreign genetic elements may be common across geographical distances.

Finally, because *P*. *agardhii* is known to be an efficient scavenger of nitrogen [11], we analyzed parts of the nitrogen uptake pathway for specific genes of interest and differences. Three isolates (1025, 1026, and 1027) contained an extra ABC-transporter for nitrate, sulfonate, and bicarbonate as well as a unique carbonate dehydrogenase (Table 5), possibly making them a better competitor for nutrients. The *nrtABCD* cluster, which encodes for a nitrate transport system, and its flanking genes, *narB*, which converts nitrate to nitrite, and *nirA*, which converts nitrite to ammonia, all contained mutations when compared to the reference sequence found in *P*. *agardhii* NIES-204. These genes are in a single operon in *P*. *agardhii* but are scattered through the genome of *Microcystis aeruginosa* [60]. While there was no difference in the *nblA* genes found across all isolates and reference sequences, there were several deletions found in the *cphA2* gene, part of the *cphBA2* operon, of 1033 and 1812 (Fig 7C). The *cphA2* gene is transcribed when nitrogen levels are low [11], and deletions in this gene may indicate ineffective or lowered affinity protein products. Further, isolate 1812 also had a different deletion in *cphA1*, the cyanophycin synthetase that is active under nitrogen replete conditions [11], making it the most divergent isolate compared to both the reference sequences and other isolates in terms of nutrient related genes.

To summarize, we present here the genomes of 20 isolates of *Planktothrix agardhii* from Sandusky Bay, a Lake Erie embayment. These genomes are closely related to each other and other isolates of the same species but display genetic variations that indicate high levels of ecological partitioning within the niche. These isolates have the genetic capabilities of producing several bioactive secondary metabolites, including microcystin congeners and two distinct classes of aeruginosides. Further, the isolates contain at least two CRISPR-cas systems, encoding for PaV-LD as well as many unknown foreign genetic elements. Additionally, genetic differences in nitrogen uptake pathways may indicate that while *P*. *agardhii* is considered a good scavenger of nitrogen, some isolates may be better scavengers than others. This work is just the first step in better understanding how *P*. *agardhii* is equipped to dominate harmful algal blooms across the globe.

## Supporting information

S1 Table Contig hit outputs for each *P*. *agardhii* isolate. (XLSX)

S2 Table Pfam hit outputs for each identifiable CDS for each *P*. *agardhii* isolate. (XLSX)

S3 Table Breakdown of taxonomic classification of non-cyanobacterial genes found in each *Planktothrix agardhii* genome. (DOCX)

S4 Table Closest related sequence for collapsed branches for secondary metabolite production. “N/A” indicates sequences missing a particular biosynthetic cluster. “***” indicates sequences that are self-represented in Fig 4.
(DOCX)

S5 Table Crispr-Cas spacer sequences for each *Planktothrix agardhii* isolate, labeled with Cas-subtype family. (XLSX)

S1 Fig Genomic rearrangement within *P*.*agardhii* tree generated groupings. (DOCX)
