# Supplementary material for: Genomic comparison of Planktothrix agardhii isolates from a Lake Erie embayment
Source: PLoS One. 2022 Aug 23;17(8):e0273454. doi: 10.1371/journal.pone.0273454 (PMC9398003; doi:10.1371/journal.pone.0273454)
Supplement: S3 Table — (DOCX) [file pone.0273454.s003.docx]

**S3 Table. Breakdown of taxonomic classification of non-cyanobacterial genes found in each *Planktothrix agardhii* genome.**

| Isolate | Protein taxonomic classification and number of hits |
| --- | --- |
| NIVA_CYA 126/8 | Bacteroidetes (6), Gammaproteobacteria- others (3), Alphaproteobacteria (3), Plantomycetes (2), Betaproteobacteria (2), Deinococcus-Thermus (1), Firmicutes – Bacilli (1), Other FCB group (1), Deferribacteres (1), Acidobacteria (1), Epsilonproteobacteria (1), Chlorobi (1) |
| 1025 | Deltaproteobacteria (9), Gammaproteobacteria- others (7), Betaproteobacteria (6), Bacteroidetes (2), Alphaproteobacteria (2), Euryarchaeota (2), Other Terrabacteria group (2), Chloroflexi (1), Firmicultes – Bacilli (1), Planctomycetes (1), Acidobacteria (1), Actinobacteria (1) |
| 1026 | Deltaproteobacteria (10), Gammaproteobacteria- others (7), Betaproteobacteria (6), Planctomycetes (4), Chloroflexi (3), Bacteroidetes (3), Alphaproteobacteria (3), Other Terrabacteria group (2), Firmicutes- Bacilli (2), Firmicutes- Clostridia (2), Acidobacteria (1), Spirochaetes (1), Atribacterota (1), Nitrospirae (1), Euryachaeota (1) |
| 1027 | Gammaproteobacteria- others (7), Planctomycetes (6), Alphaproteobacteria (5), Deltaproteobacteria (4), Betaproteobacteria (3), Bacteroidetes (3), Actinobacteria (2), Synergistetes (1), Gammaproteobacteria- Enterobacteria (1), Spirochaetes (1), Other Terrabacteria group (1) |
| 1029 | Alphaproteobacteria (9), Deltaproteobacteria (6), Gammaproteobacteria- others (6), Planctomycetes (6), Bacteroidetes (3), Betaproteobacteria (3), Chloroflexi (2), Actinobacteria (2), Other Terrabacteria group (1), Gammaproteobacteria- Enterobacteria (1), Synergistetes (1), Spirochaetes (1) |
| 1030 | Alphaproteobacteria (9), Deltaproteobacteria (6), Gammaproteobacteria- others (6), Planctomycetes (6), Bacteroidetes (3), Betaproteobacteria (3), Chloroflexi (2), Actinobacteria (2), Gammaproteobacteria- Enterobacteria (1), Spirochaetes (1), Other Terrabacteria group (1), Synergistetes (1) |
| 1031 | Bacteroidetes (7), Gammaproteobacteria- others (7), Deltaproteobacteria (5), Firmicutes- Bacilli (3), Betaproteobacteria (3), Planctomycetes (2), Actinobacteria (2), Alphaproteobacteria (1), Synergistetes (1), Gammaproteobacteria- Enterobacteria (1) |
| 1032 | Gammaproteobacteria- others (8), Deltaproteobacteria (5), Betaproteobacteria (3), Planctomycetes (3), Bacteroidetes (3), Actinobacteria (2), Firmicutes- Bacilli (2), Synergistetes (1), Alphaproteobacteria (1), Gammaproteobacteria- Enterobacteria (1) |
| 1033 | Alphaproteobacteria (13), Deltaproteobacteria (9), Betaproteobactria (6), Firmicutes- Bacilli (6), Bacteroidetes (6), Gammaproteobacteria- others (5), Firmicutes- Clostridia (4), Chloroflexi (3), Gammaproteobacteria- Enterobacteria (3), Actinobacteria (2), Planctomycetes (2), Cnidarians (1), Nitrospinia (1), Spirochaetes (1), Euryarchaeota (1), Synergistetes (1), Other FCB group (1), Verrucomicrobia (1) |
| 1801 | Bacteroidetes (6), Gammaproteobacteria- others (6), Alphaproteobacteria (5), Planctomycetes (4), Deltaproteobacteria (4), Chloroflexi (4), Betaproteobacteria (3), Firmicutes- Bacilli (2), Firmicutes- Clostridia (2), Tenericutes (2), Cadidatus Thermoplasmotota (1), Euryachaeota (1), Bacteria incertae sedis (1), Deinococcus-Thermus (1), Cnidarians (1) |
| 1803 | Bacteroidetes (6), Betaproteobacteria (5), Planctomycetes (5), Chloroflexi (4), Gammaproteobacteria- others (4), Deltaproteobacteria (2), Euryarchaeota (2) Gammaproteobacteria- Enterobacteria (2), Alphaproteobacteria (1), Actinobacteria (1), Firmicutes- Bacilli (1) |
| 1804 | Bacteroidetes (7), Planctomycetes (5), Betaproteobacteria (5), Chloroflexi (4), Gammaproteobacteria- others (3), Deltaproteobacteria (2), Euryarchaeota (2), Gammaproteobacteria- Enterobacteria (2), Alphaproteobacteria (1), Epsilonproteobacteria (1), Actinobacteria (1) |
| 1805 | Betaproteobacteria (6), Bacteroidetes (6), Planctomycetes (5), Chloroflexi(4), Gammaproteobacteria- others (4), Alphaproteobacteria (3), Euryarchaeota (2), Deltaproteobacteria (2), Gammaproteobacteria- Enterobacteria (2), Actinobacteria (1), Firmicutes- Bacilli (1) |
| 1806 | Bacteroidetes (7), Planctomycetes (5), Betaproteobacteria (5), Chloroflexi (4), Gammaproteobacteria- others (3), Gammaproteobacteria- Enterobacteria (2), Deltaproteobacteria (2), Euryarchaeota (2), Actinobacteria (1), Epsilonproteobacteria (1), Alphaproteobacteria (1) |
| 1807 | Planctomycetes (8), Bacteroidetes (7), Gammaproteobacteria- others (6), Deltaproteobacteria (5), Chloroflexi (3), Actinobacteria (3), Betaproteobacteria (3), Alphaproteobacteria (3), Euryarchaeota (2), Synergistetes (1), Gammaproteobacteria- Enterobacteria (1) |
| 1808 | Planctomycetes (8), Gammaproteobacteria- others (5), Bacteroidetes (4), Actinobacteria (3), Deltaproteobacteria (3), Alphaproteobacteria (2), Betaproteobacteria (2), Euryarchaeota (1), Synergistetes (1), Gammaproteobacteria- Enterobacteria (1) |
| 1809 | Planctomycetes (7), Gammaproteobacteria- others (6), Bacteroidetes (4), Actinobacteria (3), Alphaproteobacteria (2), Deltaproteobacteria (2), Betaproteobacteria (2), Gammaproteobacteria- Enterbacteria (1), Euryarchaeota (1) |
| 1810 | Gammaproteobacteria- others (4), Alphaproteobacteria (3), Bacteroidetes (2), Betaproteobacteria (2), Planctomycetes (1), Deltaproteobacteria (1), Verrucomicrobia (1), Spirochaetes (1), Chloroflexi (1), Synergistetes (1), Other FCB group (1) |
| 1811 | Alphaproteobacteria (65), Gammaproteobacteria- others (7), Deltaproteobacteria (5), Betaproteobacteria (5), Bacteroidetes (5), Chloroflexi (4), Euryarchaeota (3), Firmicutes- Clostridia (2), Firmicutes- Bacilli (2), Tenericutes (2), Planctomycetes (2), Gammaproteobacteria- Enterobacteria (1), Bacteria incertae sedis (1), Epsilonproteobacteria (1) |
| 1812 | Planctomycetes (9), Betaproteobacteria (9), Bacteroidetes (8), Gammaproteobacteria- others (7), Chloroflexi (6), Deltaproteobacteria (5), Alphaproteobacteria (4), Euryarchaeota (3), Firmicutes- Bacilli (3), Other Terrabacteria group (2), Tenericutes (2), Firmicutes- Clostridia (2), Actinobacteria (1), Nitrospirae (1), Gammaproteobacteria- Enterobacteria (1), Bacteria incertae sedis (1), Epsilonproteobacteria (1) |
| 1813 | Bacteroidetes (8), Gammaproteobacteria- others (7), Plactomycetes (4), Betaproteobacteria (3), Alphaproteobacteria (2), Firmicutes- Bacilli (2), Deltaproteobacteria (2), Gammaproteobacteria- Enterobacteria (2), Actinobacteria (2), Euryarchaeota (1), Epsilonproteobacteria (1) |
