# Supplementary material for: Genomic comparison of Planktothrix agardhii isolates from a Lake Erie embayment
Source: PLoS One. 2022 Aug 23;17(8):e0273454. doi: 10.1371/journal.pone.0273454 (PMC9398003; doi:10.1371/journal.pone.0273454)
Supplement: S4 Table — “N/A” indicates sequences missing a particular biosynthetic cluster. “***” indicates sequences that are self-represented in Fig 4. (DOCX) [file pone.0273454.s004.docx]

**S4 Table. Closest related sequence for collapsed branches for secondary metabolite production.** “N/A” indicates sequences missing a particular biosynthetic cluster. “***” indicates sequences that are self-represented in Figure 4.

| Isolate | Microcystin (mcy) | Aeruginosin (aer) | Anabaenapeptin (apn) | Cyanopeptin (oci) | Microviridin (mvd) | Prenylagaramide (pag) |
| --- | --- | --- | --- | --- | --- | --- |
| 1025 | N/A | *** | 1027 | 1027 | *** | 1027 |
| 1026 | *** | 1025 | 1027 | 1027 | N/A | 1027 |
| 1027 | N/A | 1025 | *** | *** | 1025 | *** |
| 1029 | 1030 | *** | 1808 | 1807 | *** | *** |
| 1030 | *** | 1029 | 1808 | 1807 | 1029 | 1029 |
| 1031 | 1030 | 1029 | 1032 | 1807 | *** | 1809 |
| 1032 | 1030 | 1029 | *** | 1807 | 1031 | 1809 |
| 1033 | *** | *** | 1810 | *** | *** | *** |
| 1801 | N/A | 1811 | N/A | *** | *** | *** |
| 1803 | N/A | *** | 1804 | 1804 | *** | 1806 |
| 1804 | N/A | 1803 | *** | *** | 1803 | 1806 |
| 1805 | N/A | 1803 | 1804 | 1804 | 1803 | 1806 |
| 1806 | N/A | 1803 | 1804 | 1804 | 1803 | *** |
| 1807 | 1030 | 1029 | 1808 | *** | 1809 | 1809 |
| 1808 | 1030 | 1029 | *** | 1807 | 1809 | 1809 |
| 1809 | 1030 | 1029 | 1808 | 1807 | *** | *** |
| 1810 | N/A | *** | *** | *** | *** | *** |
| 1811 | N/A | *** | N/A | 1812 | 1801 | *** |
| 1812 | *** | 1811 | N/A | *** | 1801 | 1811 |
| 1813 | N/A | *** | *** | *** | *** | *** |
