# Supplementary material for: Genomic comparison of Planktothrix agardhii isolates from a Lake Erie embayment
Source: PLoS One. 2022 Aug 23;17(8):e0273454. doi: 10.1371/journal.pone.0273454 (PMC9398003; doi:10.1371/journal.pone.0273454)

S1 Fig. Genomic rearrangement within *P.agardhii* tree generated groupings.

1. Group 1 (*P. agardhii* 1811, 1812 and 1801)


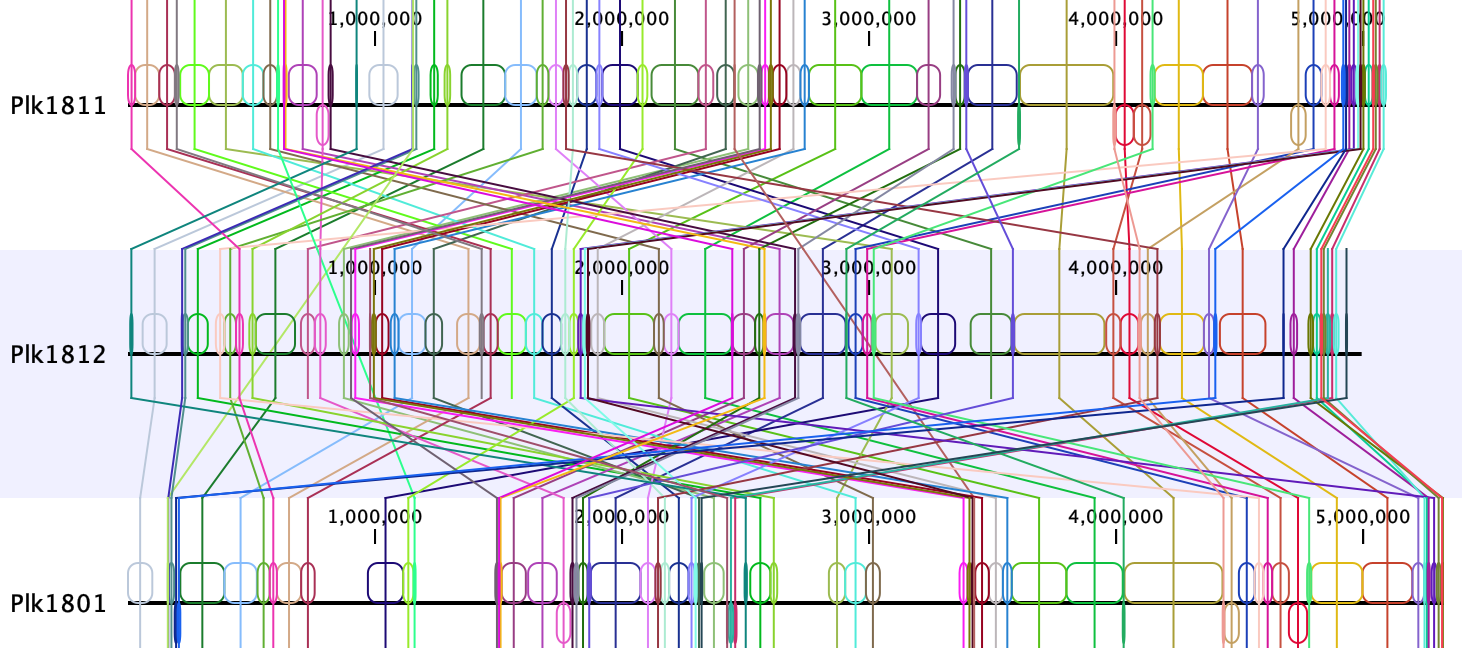


1. Group 2 (*P. agardhii* 1025, 1026, 1027, 1033, 1810 and 1813)


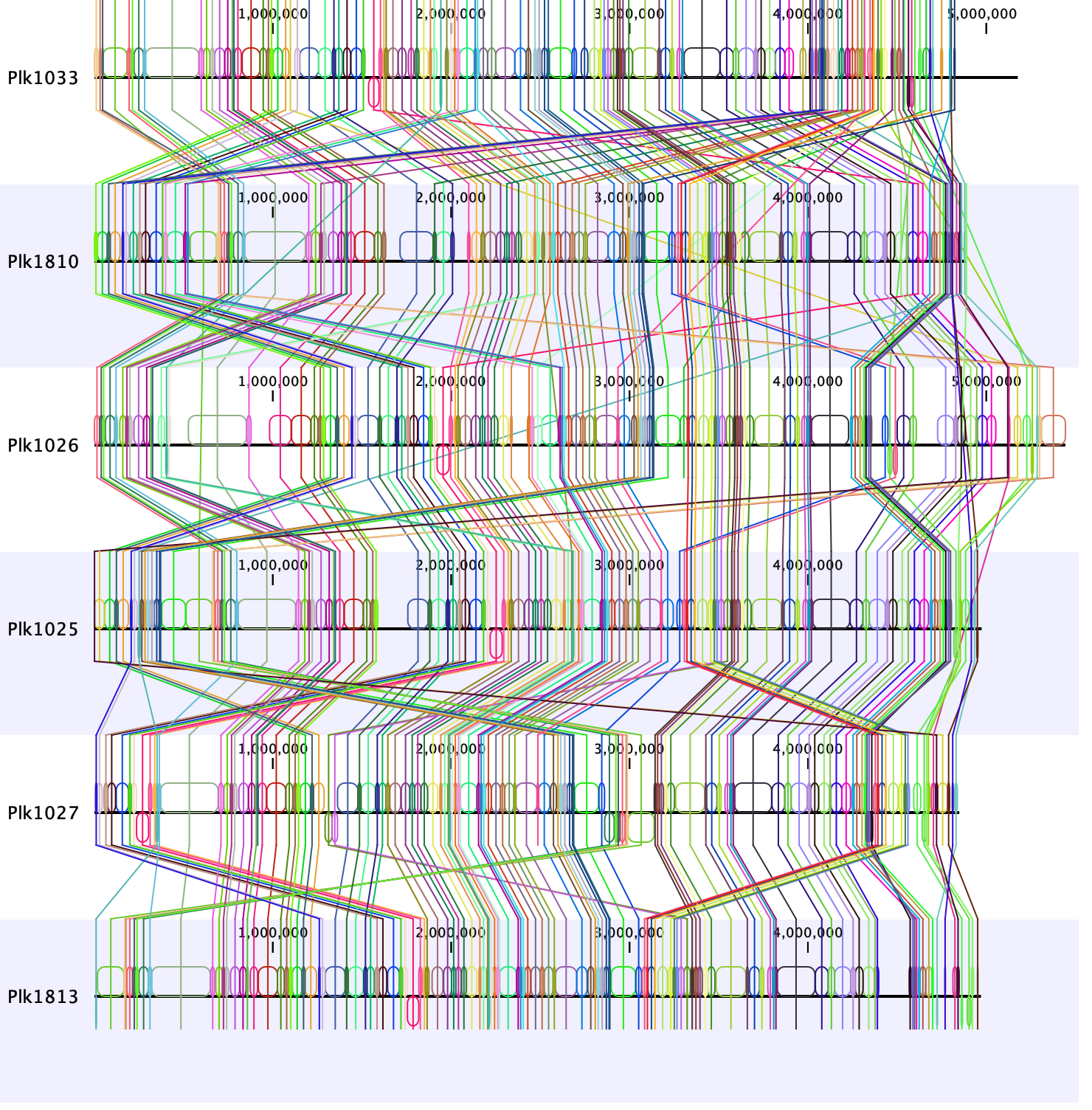


1. Group 3 (*P. agardhii* 1803, 1804, 1805 and 1806)


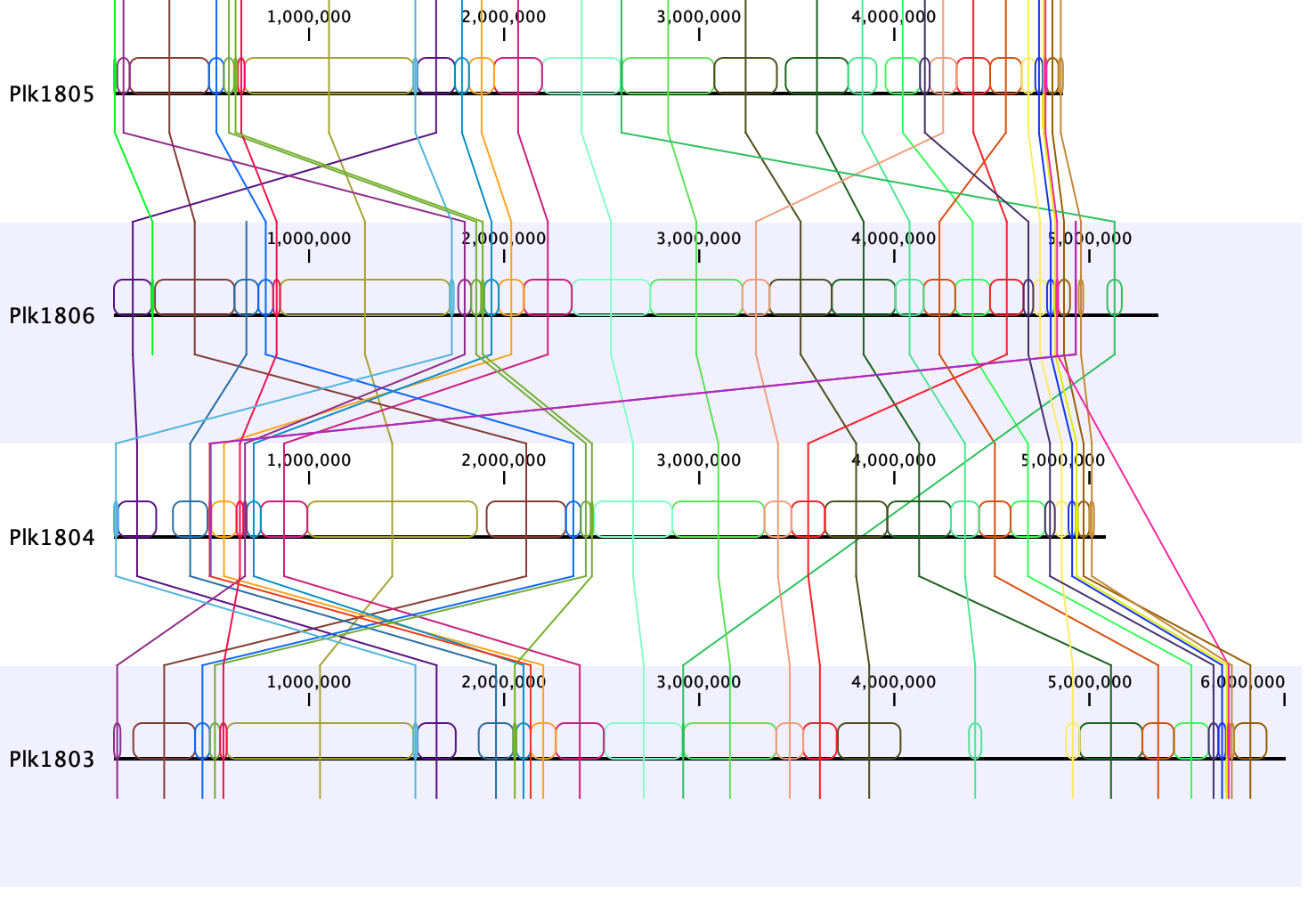


1. Group 4 (*P. agardhii* 1029, 1030, 1031, 1032, 1807, 1808 and 1809)


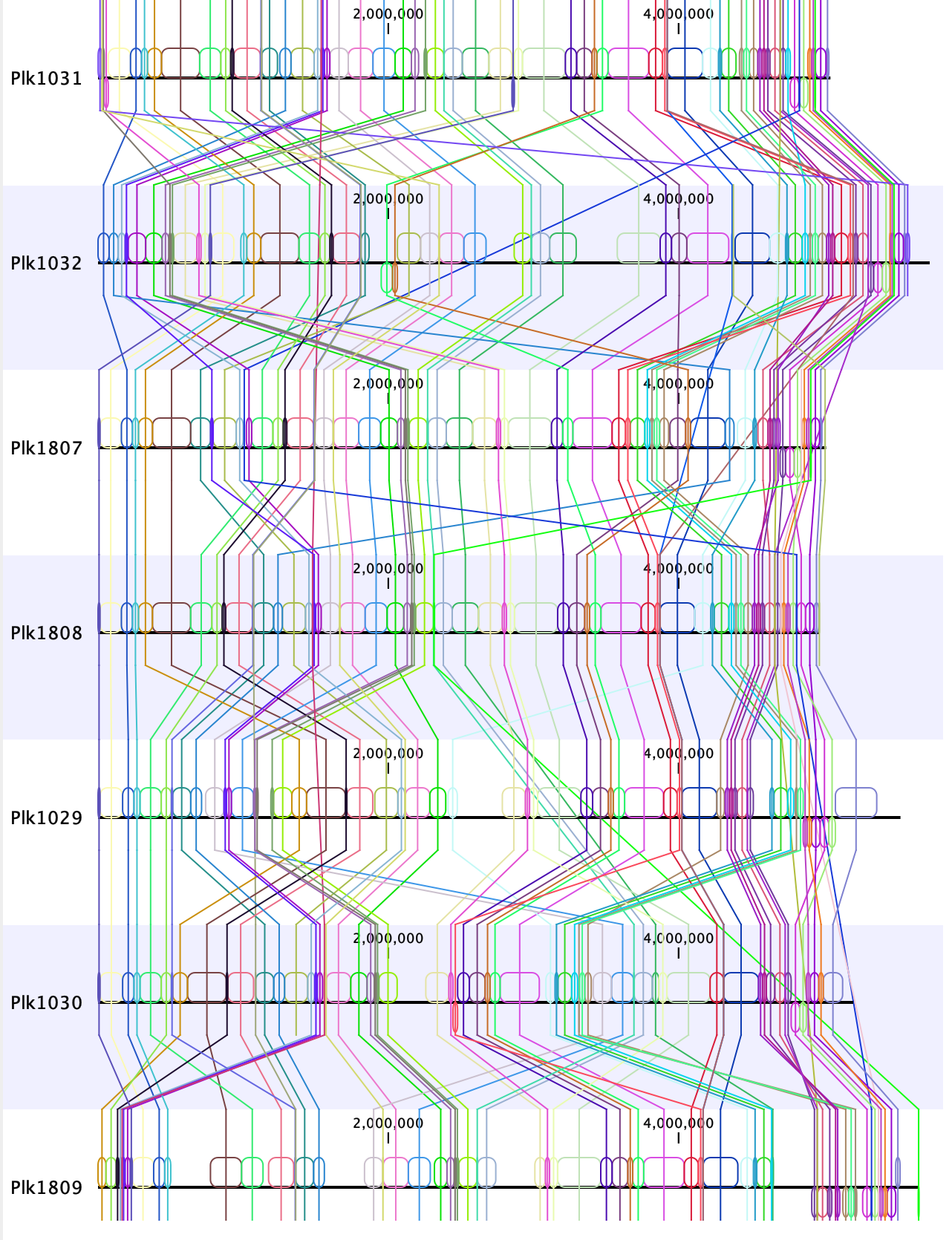

Supplement: S1 Fig — (DOCX) [file pone.0273454.s006.docx]
