# Supplementary material for: Genomic comparison of Planktothrix agardhii isolates from a Lake Erie embayment
Source: PLoS One. 2022 Aug 23;17(8):e0273454. doi: 10.1371/journal.pone.0273454 (PMC9398003; doi:10.1371/journal.pone.0273454)
Supplement: Attachment — Submitted filename: Reviewers comments.docx [file pone.0273454.s007.docx]

This paper focuses on the function and metabolic behavior of *Planktothrix agardhii* through comparative genomics research methods. As a common genus for forming harmful cyanobacterial blooms, people pay less attention to *Planktothrix* genus than *Microcystis*. The information conveyed by this article will help us better understand the role of *Planktothrix* in the formation of harmful algal blooms. This is the greatest value of this article. However, I find that this manuscript could be improved further. Generally, the logic of this article is not so clear. There are also many writing format problems in this article. The quality of the graphs and tables also needs to be improved.

The specific details are as follows:

1. Page 3 Line60 "*P. agardhii* harmful algal blooms (HABs) typically produce a suite of secondary..." may be revised to "Harmful algal blooms (HABs) typically produce a suite of secondary...".
2. Page 3 Line73 "...all of which are also found in other *P. agardhii* cultures across the globe (Kurmayer et al)" should add a comma.
3. There is too much space to introduce the MC. The second and third paragraphs may be better combined.
4. Page 9 Line 248 "(Supplemental figure 1)" to "(Supplemental Figure 1)".
5. Page 9 Line 253-255 "P. agardhii, Planktothrix" needs to be italicized.
6. Page 10 Line 276 "Supplementary table 3" may be revised to " Supplementary Table 3".
7. Page 14 Line 408-409 "P. agardhii " needs to be italicized.
8. Page 14 Line 409-410 "P. agardhii " needs to be italicized.
9. Page 26, Table1 is missing a title, and a three-line table format is recommended.
10. It is recommended to add a color legend to Figure 1.
11. The phylogenetic tree of Figure2, Figure3 and Figure5 is a bit rough and can be beautified again.
12. The Figure7 requires a uniform strain writing format.
13. The format of the figures and tables in the supplemental materials should be further checked.
