# Supplementary material for: Genomic comparison of Planktothrix agardhii isolates from a Lake Erie embayment
Source: PLoS One. 2022 Aug 23;17(8):e0273454. doi: 10.1371/journal.pone.0273454 (PMC9398003; doi:10.1371/journal.pone.0273454)
Supplement: Attachment — Submitted filename: ResponseToReviewers.docx [file pone.0273454.s008.docx]

Reviewers' comments:

Reviewer #1: This paper focuses on the function and metabolic behavior of Planktothrix agardhii through comparative genomics research methods. As a common genus for forming harmful cyanobacterial blooms, people pay less attention to Planktothrix genus than Microcystis. The information conveyed by this article will help us better understand the role of Planktothrix in the formation of harmful algal blooms. This is the greatest value of this article. However, I find that this manuscript could be improved further. Generally, the logic of this article is not so clear. There are also many writing format problems in this article. The quality of the graphs and tables also needs to be improved.
The specific details are as follows:
1.Page 3 Line60 "P. agardhii harmful algal blooms (HABs) typically produce a suite of secondary..." may be revised to "Harmful algal blooms (HABs) typically produce a suite of secondary...".

Response: The text was edited per recommendation

2.Page 3 Line73 "...all of which are also found in other P. agardhii cultures across the globe (Kurmayer et al)" should add a comma.

Response: This section was deleted.

3.There is too much space to introduce the MC. The second and third paragraphs may be better combined.

Response: The text was trimmed and combined per recommendation

4.Page 9 Line 248 "(Supplemental figure 1)" to "(Supplemental Figure 1)".

Response: The text was edited per recommendation

5.Page 9 Line 253-255 "P. agardhii, Planktothrix" needs to be italicized.

Response: The text was edited per recommendation

6.Page 10 Line 276 "Supplementary table 3" may be revised to " Supplementary Table 3".

Response: The text was edited per recommendation

7.Page 14 Line 408-409 "P. agardhii " needs to be italicized.

Response: The text was edited per recommendation

8.Page 14 Line 409-410 "P. agardhii " needs to be italicized.

Response: The text was edited per recommendation

9.Page 26, Table1 is missing a title, and a three-line table format is recommended.

Response: A title has been added to Table 1. The table was left unformatted at this time to allow for the journal to reformat per their specifications.

10.It is recommended to add a color legend to Figure 1.

Response: Grouping names were added to the tree, so a color legend should no longer be necessary

11.The phylogenetic tree of Figure2, Figure3 and Figure5 is a bit rough and can be beautified again.

Response: Trees were regenerated and saved at a higher resolution for better imagery.

12.The Figure7 requires a uniform strain writing format.

Response: The strain format is the same as figure 3, 4, and 5. Images were resampled to make them look better.

13.The format of the figures and tables in the supplemental materials should be further checked.

Response: Formatting was fixed according to journal requirements.

Reviewer #2: Overall comments:
The authors have sequenced the genomes of 20 new Planktothrix strains and performed comparative genomic analysis on them. At first glance, the manuscript appeared to present interesting results on the ecology and genomics of Planktothrix. The CRISPR-Cas system analysis in this manuscript was particularly impressive. However, some of the experimental methods are questionable, and the reliability of some of the results and discussions are also questionable. Some deficiencies were found in the descriptions regarding Figures and Tables.

Specific comments:
1) Lines 43-128. Regarding the Introduction, I felt that background and purpose of this manuscript were not well organized. For example, there is no need to indicate which strain produces which secondary metabolite, and no need to elaborate on the antifungal activity of unknown metabolite, which does not appear in the Results or Discussion at all.

Response: The introduction has been trimmed and reorganized, and the purpose of the paper more clearly lined out.

2) Lines 156-159. If the strains are non-axenic, it should be shown that the assembled genome sequences have no contaminations from other microorganisms. This point is very important to interpret the results.

Response: Taxonomic assessment of proteins was preformed using GhostKoala and non-cyanobacterial gene hits have been added to Table 1 as a percent of total genome as a proxy for potential contamination. Which genus these genes are attributed to has been added to the supplemental materials. This analysis was also performed on the reference sequence to make a comparison of non-cyanobacterial genes in the genomes.

3) Lines 176-191. If my understanding is correct, the authors have mapped the small contigs generated by de novo assembly to the reference sequence for the generation of scaffolds and large contigs. Do the sequences produced in this way reflect the true genomic arrangement? If not, how should I interpret the results of the subsequent analysis of genome rearrangement by mauve and the Average Nucleotide Identity and Alignment Percentage?

Response: The limitations of using a reference sequence for generation of scaffolds has been noted in text. In particular, the whole genome alignment requires the use of a reference genome to order the contigs into a concatenated single sequence. The use of a reference genome to generate scaffolds and the use of a reference genome to order the contigs are the same process in this case. We note that through this manipulation, some Locally Collinear Blocks may be closer together than is found in the actual sequence, which may affect alignment percentages. The ANI should not be affected by this process.

4) Regarding Figure 2, how did authors make this phylogenetic tree from the AP and ANI analysis. What does the bar with the number 0.250 mean? I could not understand Lines 235-238.

Response: The paragraph in question is the methodology used to generate the phylogenetic trees and related statistics using the CLC genomics workbench program. The ANI comparison is a tool within the program. This has been clarified within text by stating that it is a workflow and clarifying the output and parameters. The bar is the scaled length of the distance matrix which reflects the absolute distance between sequences as a function of site substitutions. This has been added to the figure legend.

5) Regarding Figure 3 and Lines 252-263, why did the authors choose the UPGMA method rather than other methods such as the maximum likelihood method to infer phylogenetic tree? It would be better to estimate the phylogenetic tree using the maximum likelihood method and show the reliability of the branches using the bootstrap method. In addition, few genes were used to estimate the phylogenetic tree, and therefore the resolution of the phylogenetic tree is very low. The authors should add more genes and increase the resolution. What does the bar with the number 0.001 in the Figure mean? Please describe it in the Figure legend.

Response: Maximum likelihood method was not available using Geneious Prime. The bar is the scaled length of the distance matrix which reflects the absolute distance between sequences. The purpose of Figure 3 was to confirm that all sequences clustered with *Planktothrix agardhii* references and not *Planktothrix rubescens* references (outgroup). With these 5 genes, we can confirm that the isolates are indeed *Planktothrix agardhii* and confirm that two of the whole genome groupings hold true. Bootstrapping has been added to the figure.

6) Lines 265-289. Why did the authors identify the genes of interest by the annotation name? Gene annotations are sometimes wrong by several reasons. If the authors discuss whether the genes exist or not in the genomes, sequence similarity search such as BLAST would be better than the gene annotation name search. It is better to explain the validity of their gene search strategy or re-examine the gene search by sequence similarity.

Response: The genes of interest were identified based on BLAST search. This was clarified in text.

7) Figure 2 and Figure 3. Topologies of the trees in Figure 2 and 3 are different. The reasons must be explained in the text.

Response: The similarities are discussed in text, particularly the last paragraph of the “General genome characteristics of Planktothrix agardhii isolates” in the results (Lines 342-351). Further text was added to explain some differences.

8) Lines 373-375 and Figure 4E. For the microviridin biosynthesis, mvdE gene is essential because the gene encodes prepeptide, but this manuscript lack this gene. The gene clusters should be compared including the gene.

Response: The analysis was rerun including mvdE gene.

9) Lines 380-384. Why did the authors concatenate the secondary metabolite gene clusters? The length of each gene cluster is different and the mutation rate seems to be different. Moreover, some strains do not have these gene clusters. I felt that the authors have not clearly explained what the phylogenetic tree means. In addition, I could not understand the sentence "Particular isolates can be chosen at random within clusters (such as 1807, 1808, or 1809), but this analysis identifies two completely unique sequences in 1033 and 1813."
10)

Response: The tree was generated to identify isolates that represent secondary metabolite diversity, as stated in the results (Lines 380-382), and added again to the methods for further clarification. To further clarify the purpose of the tree, a table of presence/absence of each gene cluster was included. The sentence in question was reworked for clarity.

Line 506. "aeruginoside 102" production by P. agardhii NIES-204 is unclear form the gene cluster similarity, and the prediction is probably wrong. Generally, substrate for NRPS should be predicted from amino acid residues in a substrate-binding pocket of its adenylation domain. Some prediction programs, such as the NRPSpredictor2 (https://github.com/roettig/NRPSpredictor2) and PKS/NRPS Analysis Web-site (http://nrps.igs.umaryland.edu) have been developed. Indeed, in my test analysis using these programs, the aeruginosin gene cluster of P. agardhii NIES-204 appeared to produce a different structure of aeruginosin than "aeruginoside 102". Additionally, Shimura et al. (Harmful Algae, 101: 101942, 2021) have analyzed the gene cluster and stated as "In P. agardhii NIES-204, aerK, which is essential for aeruginosin biosynthesis, is present as aerK1 and aerK2, lacking part of the aerK sequence. Therefore, aerK may be inactive, and P. agardhii NIES-204 may not produce aeruginosins."

Response: The information about potential production of secondary metabolites was modified to include the new reference, and left as speculation.

11) Line 65. " Dittmann et al. 1997" is not appropriate reference for the sentence because the paper only identified the microcystin gene for Microcystis. Review papers for microcystin biosynthesis may be appropriate.

Response: Additional references were added.

12) Line 68. "P. rubescens" is italic.

Response: This has been corrected.

13) Line 118. "dispersal or Ma-LMM01-like cyanophages" is probably a misspelling of "dispersal of Ma-LMM01-like cyanophages".

Response: This has been corrected.

14) Line 137. Jaworski’s Medium needs a reference.

Response: Reference has been added.

15) Line 280. Perhaps "Geneious Primer" is incorrect and "Geneious Prime" is correct.

Response: This has been corrected.

16) Lines 292-295. Gene citations should be the papers in which the genes were originally identified from cyanobacteria. If possible, INSDC Accession numbers of Ref. genes should be appended for gene identification.

Response: In the interest of keeping the paper as concise as possible, we feel adding all these requested references adds undue length to the manuscript. NCBI protein accession numbers were added to examples for each gene set, and the first description of several of the genes was added to the references.

17) Line 470. "align" is maybe "aligned".

Response: The text was edited per recommendation

18) Line 471. "cluster" is maybe "clustered"

Response: The text was edited per recommendation

19) Line 473. "separate" is maybe "separated"

Response: The text was edited per recommendation

20) Line 475. "cluster" is maybe "clustered"

Response: The text was edited per recommendation

21) Lines 497-498. I could not understand "which consisted of several mutations different than the cluster found in P. agardhii NIVA-CYA 126/8,".

Response: Text was edited for clarification.

22) Lines 501-507. The two types of aeruginosin biosynthetic gene clusters in Planktothrix have been identified and reported by Shimura et al. (Harmful Algae, 101: 101942, 2021). The authors should cite the paper.

Response: Citation has been added.

23) Figure 4. Could you explain what the box and line indicate and black and gray color means? Does the boxes represent presence of sequences and the lines represent sequence gaps? The width of the horizontal line is different in some areas. Please describe these points in the Figure legend.

Response: A color legend was added to the figure. Per the figure legend, the “Black segments in the non-highlighted sequences indicate points of difference.” The information about the grey sections and lines has been added.

24) Regarding Figure 5, description of phylogenetic analysis method is missing in the text.

Response: This information has been added to the methods.

25) Line 810. Description (title) of Table 1 is missing.

Response: A title was added.

26) Regarding Table 1, I think the number in the "No. Contig" column is incorrect. I assume from Supplementary Table 1 that it is probably the total number of contigs and scaffolds. Also, the numbers for Plk1025, Plk1031, Plk1807, Plk1808, Plk1809, Plk1810, Plk1811, and Plk1813 are 16, 30, 67, 18, 14, 19, 15, and 23, as inferred from Supplement Table 1. Please check it. If possible, the completeness of the genome sequence should be shown in Table 1.

Response: The information on select isolates was from a previous draft for Supplemental Table 1. This data has been corrected in Supplemental Table 1. The “No. Contig” title was changed to “No. contig and scaffold” for clarity.

27) Line 832. Description (title) of Table 5 is missing.

Response: A title was added

28) Regarding Table 5, how did the authors compare amt1 and amt3 genes? These genes exist as different genes but represented in one column. Did the authors concatenate the genes before comparison? Please explain about that in the text. INSDC Accession numbers of Ref. genes should be appended.

Response: These genes are linked in the Planktothrix genome, which was added to the table’s description. The Reference genes were appended with accession numbers.
