# Supplementary material for: Genomic comparison of Planktothrix agardhii isolates from a Lake Erie embayment
Source: PLoS One. 2022 Aug 23;17(8):e0273454. doi: 10.1371/journal.pone.0273454 (PMC9398003; doi:10.1371/journal.pone.0273454)
Supplement: Peer review history

# Peer review: Decision Letter 0

**DOI:** 10.1371/journal.pone.0273454.r001

**Authors:** Todd Miller

12 Apr 2022

PONE-D-22-03108Genomic comparison of Planktothrix agardhii isolates from a Lake Erie embaymentPLOS ONE

Dear Dr. Bullerjahn,

Thank you for submitting your manuscript to PLOS ONE. After careful consideration, we feel that it has merit but does not fully meet PLOS ONE’s publication criteria as it currently stands. Therefore, we invite you to submit a revised version of the manuscript that addresses the points raised during the review process. Please address the reviewers line-by-line comments. Pay attention to reviewers regarding formatting issues. In addition please address concerns regarding how toxin gene clusters were annotated. 

Please submit your revised manuscript by May 27 2022 11:59PM. If you will need more time than this to complete your revisions, please reply to this message or contact the journal office at plosone@plos.org. When you're ready to submit your revision, log on to https://www.editorialmanager.com/pone/ and select the 'Submissions Needing Revision' folder to locate your manuscript file.

Please include the following items when submitting your revised manuscript:

- A rebuttal letter that responds to each point raised by the academic editor and reviewer(s). You should upload this letter as a separate file labeled 'Response to Reviewers'.
- A marked-up copy of your manuscript that highlights changes made to the original version. You should upload this as a separate file labeled 'Revised Manuscript with Track Changes'.
- An unmarked version of your revised paper without tracked changes. You should upload this as a separate file labeled 'Manuscript'.

If you would like to make changes to your financial disclosure, please include your updated statement in your cover letter. Guidelines for resubmitting your figure files are available below the reviewer comments at the end of this letter.

If applicable, we recommend that you deposit your laboratory protocols in protocols.io to enhance the reproducibility of your results. Protocols.io assigns your protocol its own identifier (DOI) so that it can be cited independently in the future. For instructions see: https://journals.plos.org/plosone/s/submission-guidelines#loc-laboratory-protocols. Additionally, PLOS ONE offers an option for publishing peer-reviewed Lab Protocol articles, which describe protocols hosted on protocols.io. Read more information on sharing protocols at https://plos.org/protocols?utm_medium=editorial-email&utm_source=authorletters&utm_campaign=protocols.

We look forward to receiving your revised manuscript.

Kind regards,

Todd Miller, PhD

Academic Editor

PLOS ONE

Journal Requirements:1. When submitting your revision, we need you to address these additional requirements. Please ensure that your manuscript meets PLOS ONE's style requirements, including those for file naming. The PLOS ONE style templates can be found at https://journals.plos.org/plosone/s/file?id=wjVg/PLOSOne_formatting_sample_main_body.pdf and https://journals.plos.org/plosone/s/file?id=ba62/PLOSOne_formatting_sample_title_authors_affiliations.pdf 2. Thank you for stating in your Funding Statement: (This work was supported by funding from the Ohio Department of Natural Resources [GSB], the Natural Sciences and Engineering Research Council of Canada [RMM] and National Institutes of Health [1P01ES028939-01] and National Science Foundation [OCE-1840715] awards to the Bowling Green State University Great Lakes Center for Fresh Waters and Human Health [GSB, RMM]. The funders had no role in study design, data collection and analysis, decision to publish, or preparation of the manuscript.) Please provide an amended statement that declares *all* the funding or sources of support (whether external or internal to your organization) received during this study, as detailed online in our guide for authors at http://journals.plos.org/plosone/s/submit-now.  Please also include the statement “There was no additional external funding received for this study.” in your updated Funding Statement. Please include your amended Funding Statement within your cover letter. We will change the online submission form on your behalf." 3. Thank you for stating the following in the Acknowledgments Section of your manuscript: (We thank the Ohio Department of Natural Resources for providing access to boat time for sampling in Sandusky Bay, and the members of the Bullerjahn, McKay, and Davis labs for collecting water samples. We would also like to thank Dr. Rainer Kurmayer for his support with the isolation of 2016 Planktothrix agardhii isolates as well as providing feedback on this manuscript.  This work was supported by funding from the Ohio Department of Natural Resources [GSB], the Natural Sciences and Engineering Research Council of Canada [RMM] and National Institutes of Health [1P01ES028939-01] and National Science Foundation [OCE-1840715] awards to the Bowling Green State University Great Lakes Center for Fresh Waters and Human Health [GSB, RMM].) We note that you have provided funding information that is not currently declared in your Funding Statement. However, funding information should not appear in the Acknowledgments section or other areas of your manuscript. We will only publish funding information present in the Funding Statement section of the online submission form. Please remove any funding-related text from the manuscript and let us know how you would like to update your Funding Statement. Currently, your Funding Statement reads as follows: (This work was supported by funding from the Ohio Department of Natural Resources [GSB], the Natural Sciences and Engineering Research Council of Canada [RMM] and National Institutes of Health [1P01ES028939-01] and National Science Foundation [OCE-1840715] awards to the Bowling Green State University Great Lakes Center for Fresh Waters and Human Health [GSB, RMM]. The funders had no role in study design, data collection and analysis, decision to publish, or preparation of the manuscript.) 4. Please include your amended statements within your cover letter; we will change the online submission form on your behalf.Please review your reference list to ensure that it is complete and correct. If you have cited papers that have been retracted, please include the rationale for doing so in the manuscript text, or remove these references and replace them with relevant current references. Any changes to the reference list should be mentioned in the rebuttal letter that accompanies your revised manuscript. If you need to cite a retracted article, indicate the article’s retracted status in the References list and also include a citation and full reference for the retraction notice.

[Note: HTML markup is below. Please do not edit.]

Reviewers' comments:

Reviewer's Responses to Questions

**Comments to the Author**

1. Is the manuscript technically sound, and do the data support the conclusions?

The manuscript must describe a technically sound piece of scientific research with data that supports the conclusions. Experiments must have been conducted rigorously, with appropriate controls, replication, and sample sizes. The conclusions must be drawn appropriately based on the data presented. 

Reviewer #1: Yes

Reviewer #2: Partly

**********

2. Has the statistical analysis been performed appropriately and rigorously? 

Reviewer #1: Yes

Reviewer #2: I Don't Know

**********

3. Have the authors made all data underlying the findings in their manuscript fully available?

The PLOS Data policy requires authors to make all data underlying the findings described in their manuscript fully available without restriction, with rare exception (please refer to the Data Availability Statement in the manuscript PDF file). The data should be provided as part of the manuscript or its supporting information, or deposited to a public repository. For example, in addition to summary statistics, the data points behind means, medians and variance measures should be available. If there are restrictions on publicly sharing data—e.g. participant privacy or use of data from a third party—those must be specified.

Reviewer #1: Yes

Reviewer #2: Yes

**********

4. Is the manuscript presented in an intelligible fashion and written in standard English?

PLOS ONE does not copyedit accepted manuscripts, so the language in submitted articles must be clear, correct, and unambiguous. Any typographical or grammatical errors should be corrected at revision, so please note any specific errors here.

Reviewer #1: Yes

Reviewer #2: Yes

**********

5. Review Comments to the Author

Please use the space provided to explain your answers to the questions above. You may also include additional comments for the author, including concerns about dual publication, research ethics, or publication ethics. (Please upload your review as an attachment if it exceeds 20,000 characters)

Reviewer #1: This paper focuses on the function and metabolic behavior of Planktothrix agardhii through comparative genomics research methods. As a common genus for forming harmful cyanobacterial blooms, people pay less attention to Planktothrix genus than Microcystis. The information conveyed by this article will help us better understand the role of Planktothrix in the formation of harmful algal blooms. This is the greatest value of this article. However, I find that this manuscript could be improved further. Generally, the logic of this article is not so clear. There are also many writing format problems in this article. The quality of the graphs and tables also needs to be improved.

The specific details are as follows:

1.Page 3 Line60 "P. agardhii harmful algal blooms (HABs) typically produce a suite of secondary..." may be revised to "Harmful algal blooms (HABs) typically produce a suite of secondary...".

2.Page 3 Line73 "...all of which are also found in other P. agardhii cultures across the globe (Kurmayer et al)" should add a comma.

3.There is too much space to introduce the MC. The second and third paragraphs may be better combined.

4.Page 9 Line 248 "(Supplemental figure 1)" to "(Supplemental Figure 1)".

5.Page 9 Line 253-255 "P. agardhii, Planktothrix" needs to be italicized.

6.Page 10 Line 276 "Supplementary table 3" may be revised to " Supplementary Table 3".

7.Page 14 Line 408-409 "P. agardhii " needs to be italicized.

8.Page 14 Line 409-410 "P. agardhii " needs to be italicized.

9.Page 26, Table1 is missing a title, and a three-line table format is recommended.

10.It is recommended to add a color legend to Figure 1.

11.The phylogenetic tree of Figure2, Figure3 and Figure5 is a bit rough and can be beautified again.

12.The Figure7 requires a uniform strain writing format.

13.The format of the figures and tables in the supplemental materials should be further checked.

Reviewer #2: Overall comments:

The authors have sequenced the genomes of 20 new Planktothrix strains and performed comparative genomic analysis on them. At first glance, the manuscript appeared to present interesting results on the ecology and genomics of Planktothrix. The CRISPR-Cas system analysis in this manuscript was particularly impressive. However, some of the experimental methods are questionable, and the reliability of some of the results and discussions are also questionable. Some deficiencies were found in the descriptions regarding Figures and Tables.

Specific comments:

1) Lines 43-128. Regarding the Introduction, I felt that background and purpose of this manuscript were not well organized. For example, there is no need to indicate which strain produces which secondary metabolite, and no need to elaborate on the antifungal activity of unknown metabolite, which does not appear in the Results or Discussion at all.

2) Lines 156-159. If the strains are non-axenic, it should be shown that the assembled genome sequences have no contaminations from other microorganisms. This point is very important to interpret the results.

3) Lines 176-191. If my understanding is correct, the authors have mapped the small contigs generated by de novo assembly to the reference sequence for the generation of scaffolds and large contigs. Do the sequences produced in this way reflect the true genomic arrangement? If not, how should I interpret the results of the subsequent analysis of genome rearrangement by mauve and the Average Nucleotide Identity and Alignment Percentage?

4) Regarding Figure 2, how did authors make this phylogenetic tree from the AP and ANI analysis. What does the bar with the number 0.250 mean? I could not understand Lines 235-238.

5) Regarding Figure 3 and Lines 252-263, why did the authors choose the UPGMA method rather than other methods such as the maximum likelihood method to infer phylogenetic tree? It would be better to estimate the phylogenetic tree using the maximum likelihood method and show the reliability of the branches using the bootstrap method. In addition, few genes were used to estimate the phylogenetic tree, and therefore the resolution of the phylogenetic tree is very low. The authors should add more genes and increase the resolution. What does the bar with the number 0.001 in the Figure mean? Please describe it in the Figure legend.

6) Lines 265-289. Why did the authors identify the genes of interest by the annotation name? Gene annotations are sometimes wrong by several reasons. If the authors discuss whether the genes exist or not in the genomes, sequence similarity search such as BLAST would be better than the gene annotation name search. It is better to explain the validity of their gene search strategy or re-examine the gene search by sequence similarity.

7) Figure 2 and Figure 3. Topologies of the trees in Figure 2 and 3 are different. The reasons must be explained in the text.

8) Lines 373-375 and Figure 4E. For the microviridin biosynthesis, mvdE gene is essential because the gene encodes prepeptide, but this manuscript lack this gene. The gene clusters should be compared including the gene.

9) Lines 380-384. Why did the authors concatenate the secondary metabolite gene clusters? The length of each gene cluster is different and the mutation rate seems to be different. Moreover, some strains do not have these gene clusters. I felt that the authors have not clearly explained what the phylogenetic tree means. In addition, I could not understand the sentence "Particular isolates can be chosen at random within clusters (such as 1807, 1808, or 1809), but this analysis identifies two completely unique sequences in 1033 and 1813."

10) Line 506. "aeruginoside 102" production by P. agardhii NIES-204 is unclear form the gene cluster similarity, and the prediction is probably wrong. Generally, substrate for NRPS should be predicted from amino acid residues in a substrate-binding pocket of its adenylation domain. Some prediction programs, such as the NRPSpredictor2 (https://github.com/roettig/NRPSpredictor2) and PKS/NRPS Analysis Web-site (http://nrps.igs.umaryland.edu) have been developed. Indeed, in my test analysis using these programs, the aeruginosin gene cluster of P. agardhii NIES-204 appeared to produce a different structure of aeruginosin than "aeruginoside 102". Additionally, Shimura et al. (Harmful Algae, 101: 101942, 2021) have analyzed the gene cluster and stated as "In P. agardhii NIES-204, aerK, which is essential for aeruginosin biosynthesis, is present as aerK1 and aerK2, lacking part of the aerK sequence. Therefore, aerK may be inactive, and P. agardhii NIES-204 may not produce aeruginosins."

11) Line 65. " Dittmann et al. 1997" is not appropriate reference for the sentence because the paper only identified the microcystin gene for Microcystis. Review papers for microcystin biosynthesis may be appropriate.

12) Line 68. "P. rubescens" is italic.

13) Line 118. "dispersal or Ma-LMM01-like cyanophages" is probably a misspelling of "dispersal of Ma-LMM01-like cyanophages".

14) Line 137. Jaworski’s Medium needs a reference.

15) Line 280. Perhaps "Geneious Primer" is incorrect and "Geneious Prime" is correct.

16) Lines 292-295. Gene citations should be the papers in which the genes were originally identified from cyanobacteria. If possible, INSDC Accession numbers of Ref. genes should be appended for gene identification.

17) Line 470. "align" is maybe "aligned".

18) Line 471. "cluster" is maybe "clustered"

19) Line 473. "separate" is maybe "separated"

20) Line 475. "cluster" is maybe "clustered"

21) Lines 497-498. I could not understand "which consisted of several mutations different than the cluster found in P. agardhii NIVA-CYA 126/8,".

22) Lines 501-507. The two types of aeruginosin biosynthetic gene clusters in Planktothrix have been identified and reported by Shimura et al. (Harmful Algae, 101: 101942, 2021). The authors should cite the paper.

23) Figure 4. Could you explain what the box and line indicate and black and gray color means? Does the boxes represent presence of sequences and the lines represent sequence gaps? The width of the horizontal line is different in some areas. Please describe these points in the Figure legend.

24) Regarding Figure 5, description of phylogenetic analysis method is missing in the text.

25) Line 810. Description (title) of Table 1 is missing.

26) Regarding Table 1, I think the number in the "No. Contig" column is incorrect. I assume from Supplementary Table 1 that it is probably the total number of contigs and scaffolds. Also, the numbers for Plk1025, Plk1031, Plk1807, Plk1808, Plk1809, Plk1810, Plk1811, and Plk1813 are 16, 30, 67, 18, 14, 19, 15, and 23, as inferred from Supplement Table 1. Please check it. If possible, the completeness of the genome sequence should be shown in Table 1.

27) Line 832. Description (title) of Table 5 is missing.

28) Regarding Table 5, how did the authors compare amt1 and amt3 genes? These genes exist as different genes but represented in one column. Did the authors concatenate the genes before comparison? Please explain about that in the text. INSDC Accession numbers of Ref. genes should be appended.

**********

6. PLOS authors have the option to publish the peer review history of their article (what does this mean?). If published, this will include your full peer review and any attached files.

If you choose “no”, your identity will remain anonymous but your review may still be made public.

**Do you want your identity to be public for this peer review?** For information about this choice, including consent withdrawal, please see our Privacy Policy.

Reviewer #1: No

Reviewer #2: No

[NOTE: If reviewer comments were submitted as an attachment file, they will be attached to this email and accessible via the submission site. Please log into your account, locate the manuscript record, and check for the action link "View Attachments". If this link does not appear, there are no attachment files.]

While revising your submission, please upload your figure files to the Preflight Analysis and Conversion Engine (PACE) digital diagnostic tool, https://pacev2.apexcovantage.com/. PACE helps ensure that figures meet PLOS requirements. To use PACE, you must first register as a user. Registration is free. Then, login and navigate to the UPLOAD tab, where you will find detailed instructions on how to use the tool. If you encounter any issues or have any questions when using PACE, please email PLOS at figures@plos.org. Please note that Supporting Information files do not need this step.

Attachment Submitted filename: Reviewers comments.docx Click here for additional data file.

# Author comment: Author response to Decision Letter 0

**DOI:** 10.1371/journal.pone.0273454.r002

10 Jun 2022

Responses to reviewers are provided as a separate file

Attachment Submitted filename: ResponseToReviewers.docx Click here for additional data file.

# Editor report: Decision Letter 1

**DOI:** 10.1371/journal.pone.0273454.r003

**Authors:** Todd Miller

9 Aug 2022

Genomic comparison of Planktothrix agardhii isolates from a Lake Erie embayment

PONE-D-22-03108R1

Dear Dr. Bullerjahn,

We’re pleased to inform you that your manuscript has been judged scientifically suitable for publication and will be formally accepted for publication once it meets all outstanding technical requirements.

Within one week, you’ll receive an e-mail detailing the required amendments. When these have been addressed, you’ll receive a formal acceptance letter and your manuscript will be scheduled for publication.

An invoice for payment will follow shortly after the formal acceptance. To ensure an efficient process, please log into Editorial Manager at http://www.editorialmanager.com/pone/, click the 'Update My Information' link at the top of the page, and double check that your user information is up-to-date. If you have any billing related questions, please contact our Author Billing department directly at authorbilling@plos.org.

If your institution or institutions have a press office, please notify them about your upcoming paper to help maximize its impact. If they’ll be preparing press materials, please inform our press team as soon as possible -- no later than 48 hours after receiving the formal acceptance. Your manuscript will remain under strict press embargo until 2 pm Eastern Time on the date of publication. For more information, please contact onepress@plos.org.

Kind regards,

Todd Miller, PhD

Academic Editor

PLOS ONE

Additional Editor Comments (optional):

Reviewers' comments:

# Editor report: Acceptance letter

**DOI:** 10.1371/journal.pone.0273454.r004

**Authors:** Todd Miller

12 Aug 2022

PONE-D-22-03108R1 

Genomic comparison of *Planktothrix agardhii* isolates from a Lake Erie embayment 

Dear Dr. Bullerjahn:

I'm pleased to inform you that your manuscript has been deemed suitable for publication in PLOS ONE. Congratulations! Your manuscript is now with our production department. 

If your institution or institutions have a press office, please let them know about your upcoming paper now to help maximize its impact. If they'll be preparing press materials, please inform our press team within the next 48 hours. Your manuscript will remain under strict press embargo until 2 pm Eastern Time on the date of publication. For more information please contact onepress@plos.org.

If we can help with anything else, please email us at plosone@plos.org. 

Thank you for submitting your work to PLOS ONE and supporting open access. 

Kind regards, 

PLOS ONE Editorial Office Staff

on behalf of

Dr. Todd Miller 

Academic Editor

PLOS ONE
